# Optimal Two‐Phase Sampling Designs for Generalized Raking Estimators With Multiple Parameters of Interest

**DOI:** 10.1002/sim.70786

**Published:** 2026-10-16

**Authors:** Jasper B. Yang, Bryan E. Shepherd, Thomas Lumley, Pamela A. Shaw

**Affiliations:** ^1^ Department of Biostatistics University of Washington Seattle Washington USA; ^2^ Department of Biostatistics Vanderbilt University Nashville Tennessee USA; ^3^ Department of Statistics University of Auckland Auckland New Zealand; ^4^ Biostatistics Division Kaiser Permanente Washington Health Research Institute Seattle Washington USA

**Keywords:** A‐optimality, generalized raking, measurement error, Neyman allocation, two‐phase sampling

## Abstract

Large observational datasets, including those derived from electronic health records, are a valuable resource for medical research but are often affected by missingness, measurement error, and misclassification. Two‐phase sampling with generalized raking (GR) estimation is an efficient and robust approach to statistical inference in such settings. In this approach, variables that are unavailable or measured with error in a large Phase 1 cohort are obtained with higher‐quality measurements in a Phase 2 subsample. Previous research has studied optimal Phase 2 sampling designs for inverse‐probability‐weighted (IPW) estimators in nonadaptive, multiparameter settings, and for GR estimators in single‐parameter settings. In this work, we extend these results by deriving optimal adaptive, multiwave sampling designs for IPW and GR estimators when multiple parameters are of interest. We propose several practical allocation strategies, examine the design choices that arise when implementing them, and evaluate their performance through extensive simulations and an example using data from people living with HIV attending the Vanderbilt Comprehensive Care Clinic (VCCC). Our results show that independently optimizing allocation for each parameter improves efficiency over traditional case‐control sampling. We also derive an integer‐valued, A‐optimal allocation method that typically outperforms independent optimization. Notably, we find that optimal designs for GR can differ substantially from those for IPW, and that this distinction can meaningfully affect estimator efficiency in the multiple‐parameter setting. These findings offer practical guidance for future two‐phase studies involving incomplete or error‐prone data.

## 1 Introduction

Routinely collected observational data are an increasingly popular resource for biomedical research, largely due to the widespread adoption of electronic health records (EHRs) [1]. This is a promising trend, as EHR databases hold clinically relevant information for large cohorts and are cost‐effective compared to traditional study data. However, because these data were not originally collected for research purposes, they often lack variables of interest, and the variables that are available are susceptible to measurement error and misclassification [2, 3]. Neglecting these issues can lead to misleading research findings, which may in turn negatively affect patient health outcomes [4, 5].

A common approach in these settings is to supplement the initial dataset with a validation study where error‐free and otherwise expensive measures are collected for a subset of the initial cohort. This approach can be viewed through the lens of two‐phase sampling, where the inexpensive data are collected in Phase 1, and the validation subset is gathered in Phase 2 [6, 7, 8]. To estimate parameters efficiently under two‐phase sampling, two key components must be addressed: estimation and design. Estimation approaches have been well‐studied in this context and can generally be categorized as model‐based or design‐based. The latter, which includes Horvitz–Thompson inverse‐probability‐weighted (IPW) estimators and the generally more efficient augmented inverse‐probability‐weighted (AIPW) estimators, are often preferred due to their robust properties [9, 10, 11, 12]. The second key component, design, is the focus of this work. In particular, we focus on the optimal design of Phase 2 with respect to design‐based estimators.

It is well known that stratified sampling yields efficient design‐based estimators when individuals differ across readily available variables [13, 14]. Under stratified sampling, Neyman [15] showed that the variance of a population total (or mean) is approximately minimized when stratum sample sizes are proportional to the product of a stratum's standard deviation and size, and Wright [16] later presented an exactly optimal algorithm. Breslow et al. [17] extended this idea to regression coefficients by harnessing their asymptotic equivalence to totals of influence functions, and Chen and Lumley [18] further extended this work for generalized raking (GR) estimators. Although the true influence functions for the population cannot be obtained in practice, a benefit of two‐phase sampling is that they can be estimated from Phase 1 data. Noting that the accuracy of this estimation will depend on the (unknown) correlation between the available Phase 1 variables and the expensive Phase 2 variables, some have proposed breaking up Phase 2 sampling into multiple waves so that data collected in prior waves can be used to inform the design of subsequent waves [19, 20]. Studies applying these multiwave methods have already been conducted with promising results [8].

The aforementioned research on two‐phase designs has largely focused on maximizing efficiency with respect to a single parameter, but there is an emerging need for further understanding of efficiency with respect to multiple parameters in a single study. Recently, Sauer et al. [21] and Rivera‐Rodriguez et al. [22] proposed methods for optimizing two‐phase stratified sampling for multiple parameters in cluster‐correlated settings, focusing on minimizing the weighted sum of variances, and Wang et al. [23] and Yu et al. [24] used a similar approach to address computational issues in large‐scale estimation problems. These works acknowledge the challenge of estimating optimality parameters and incorporate pilot Phase 2 samples in their examples; however, they do not fully investigate multiwave approaches, which allow for better estimation of design nuisance parameters by re‐estimating them as validation data accumulate. Additionally, their methods focus on optimal designs for IPW estimators, and their performance has not been evaluated alongside more efficient approaches such as GR and its close relative, AIPW. In a related work, Wang et al. [25] introduced a model‐free approach for approximating optimal designs for multiple parameters under a maximin criterion but did not consider multiwave sampling, and their focus on Poisson sampling contrasts with the stratified sampling designs considered here. Finally, similar ideas have also recently appeared in the active statistical inference and prediction‐powered inference literatures, where predictions are used to guide the collection of costly labeling data to improve downstream estimation efficiency [26, 27, 28]. These methods do not generally use stratified sampling or GR, are not typically framed as measurement error problems from a survey sampling perspective, and do not focus on the practical design considerations that arise when multiple parameters are of interest.

In practice, multiwave sampling with GR has been applied to multiparameter problems, but often in ad hoc ways. For example, Shepherd et al. [8] examined the association between maternal weight change during pregnancy and two outcomes, childhood obesity and asthma, using a two‐phase, multiwave design. Their sampling was optimized separately by outcome, first allocating 75% of the Phase 2 sample for estimation of the primary obesity parameter and the remaining samples for the secondary asthma parameter. It is unlikely that this strategy was truly optimal for the research goals. There is large potential for other scenarios where researchers may want to answer multiple questions in one study, so understanding optimal design for these cases is needed.

In this paper, we propose practical strategies for optimal stratified sampling in two‐phase, multiwave studies with multiple parameters of interest and examine the methodological considerations that arise in doing so. These strategies include a simultaneous approach where optimum allocation is computed at each wave for every parameter of interest, a sequential approach where optimum allocation is computed at each wave for one parameter of interest, and an A‐optimality approach where optimum allocation is computed at each wave to minimize the sum of variances for the multiple‐parameter estimates of interest. We also derive a novel extension of Wright's exact integer‐valued allocation for one parameter to A‐optimal allocation for multiple parameters. The rest of the paper is organized as follows. In Section 2 we review optimal design results when one parameter is of interest. In Section 3, we extend these results to the multiple‐parameter case and develop the three proposed strategies. These strategies are compared in a simulation study in Section 4 and are demonstrated in an example from the Vanderbilt Comprehensive Care Clinic (VCCC) HIV Study in Section 5. We conclude with summaries of our findings and recommendations for future studies.

## 2 Optimal Two‐Phase Study Designs for One Parameter

### 2.1 Design‐Based Estimators

Design‐based estimation is a robust approach to population inference that focuses on randomness due to the sampling process rather than the data‐generating mechanism. A canonical example is the Horvitz–Thompson IPW estimator for a population total $T_{y}=\sum_{i=1}^{N}y_{i}$ using a sample of size n. The IPW estimator in this case is ${\hat{T}}_{IPW}=\sum_{i=1}^{N}R_{i}\pi_{i}^{-1}y_{i}$, where $R_{i}$ is the indicator for whether unit i is sampled (so that $\sum_{i=1}^{N}R_{i}=n$) and the weight $\pi_{i}^{-1}:=w_{i}$ is the inverse of $\pi_{i}$, the sampling probability of unit i. This estimator is unbiased for $T_{y}$ provided that the $\pi_{i}$'s are nonzero, and it requires no distributional assumptions on the $y_{i}$'s.

In practice, the collection of $y_{i}$ on a sample of size n is often supplemented with less expensive auxiliary information, denoted $c_{i}$, consisting of one or more variables correlated with y and available for the entire population of size N. In such cases, another design‐based estimation approach is GR, which uses the known population total of $c_{i}$ to improve IPW estimators for the total of $y_{i}$ [29]. This improvement is achieved by calibrating sampling weights so that the weighted estimate of the population total for the auxiliary variable using the subsample matches the true population total. More formally, GR estimators adjust IPW weights by a factor of $g_{i}$ so that they become $w_{i}^{*}=g_{i}\pi_{i}^{-1}$ for individual i, and the estimator becomes ${\hat{T}}_{\text{GR}}=\sum_{i=1}^{N}R_{i}w_{i}^{*}y_{i},$ where the $g_{i}$ values are chosen such that

(1) $$\overset{N}{\underset{i=1}{\sum }}R_{i}w_{i}^{*}c_{i}=\overset{N}{\underset{i=1}{\sum }}c_{i}.$$

Since there may be multiple values of $g_{i}$ which satisfy Equation (1), the specific $g_{i}$ values are selected as those that minimize $\sum_{i=1}^{N}R_{i}d(g_{i}/\pi_{i},1/\pi_{i})$ for distance measure d(a,b)=alog(a/b)−a+b. GR estimators are at least as asymptotically efficient as traditional IPW estimators and are more efficient when components of the calibration (or raking) variable c are correlated with the variable of interest [17]. Moreover, GR estimators are consistent under the same assumptions as IPW estimators and straightforward to implement in standard statistical software, so these efficiency gains come at little additional cost [30].

Compared to a finite population total, a more general target of estimation is an arbitrary scalar super‐population parameter β. In this work, we consider settings where an ideal estimator $\tilde{\beta }$ for β would be available if the variables collected in Phase 2 (e.g., validation study) were available for the entire Phase 1 cohort. Further, we assume that $\tilde{\beta }$ is asymptotically linear, meaning it admits the form

(2) $$\sqrt{N}(\tilde{\beta }-\beta )=\frac{1}{\sqrt{N}}\overset{N}{\underset{i=1}{\sum }}h_{i}+o_{p}(1),$$

where $h_{i}$ is the influence function for unit i evaluated at the true β. Asymptotic linearity holds under standard regularity conditions for many commonly used estimators, including estimators of coefficients in generalized linear and Cox regression models. Accordingly, $\tilde{\beta }$ can be expressed asymptotically as $\beta +\frac{1}{N}\sum_{i=1}^{N}h_{i}$, so $\tilde{\beta }$ is asymptotically equivalent to a constant plus a sum of influence functions. An IPW Phase 2 estimator ${\hat{\beta }}_{\text{IPW}}$ can in turn be represented by

(3) $$\sqrt{N}({\hat{\beta }}_{\text{IPW}}-\beta )=\frac{1}{\sqrt{N}}\overset{N}{\underset{i=1}{\sum }}R_{i}\pi_{i}^{-1}h_{i}+o_{p}(1),$$

under the asymptotic regime where $\pi_{i}>\delta >0$, N→∞, n→∞ and n/N→γ∈(0,1).

The form of a GR estimator ${\hat{\beta }}_{\text{GR}}$ follows similarly to Equation (3), with weights $\pi_{i}^{-1}$ replaced by calibrated weights satisfying Equation (1). In this setting, $c_{i}$ contains information available for the entire Phase 1 cohort. Breslow et al. [17] showed that the optimal c, denoted $c^{\text{opt}}$, is the conditional expectation of $h_{i}$ given the observed Phase 1 data. GR estimators calibrated in this way are asymptotically efficient among design‐based estimators and asymptotically equivalent to the optimal augmented IPW (AIPW) estimator of Robins et al. [31] and Lumley et al. [32]. In practice, $c^{\text{opt}}$ is unknown but can be estimated through regression or imputation [11, 33, 34, 35].

### 2.2 Optimal Designs for IPW Estimators: Neyman Allocation

A key feature of the two‐phase setting is the ability to control the Phase 2 sampling design. It is well known in the survey literature that informed design choices can reduce the variance of estimators of finite population means, and the setup described in Section 2.1 illustrates that the same ideas can be applied to design‐based estimators of superpopulation parameters. This connection is made clear by noting that $\tilde{\beta }$ and the influence functions $h_{i}$ are fixed, though unknown, quantities conditional on the realized Phase 1 sample. Thus, $\tilde{\beta }$ can be viewed as a finite‐population target parameter from a Phase 2 perspective, and the design goal is hence to minimize the conditional variance of ${\hat{\beta }}_{\text{IPW}}$ or ${\hat{\beta }}_{\text{GR}}$. Reducing this conditional variance also reduces the overall variance of ${\hat{\beta }}_{\text{IPW}}$ or ${\hat{\beta }}_{\text{GR}}$, while the variation arising from Phase 1 is unaffected by the Phase 2 design. In the remainder of this work, we use this perspective to motivate optimal design approaches, focusing on stratified sampling, which has been shown to be efficient in two‐phase studies [14, 36].

Under stratified sampling with simple random sampling within strata, the population is partitioned into K strata of size $N_{k}$, and a sample of size $n_{k}$ is drawn uniformly without replacement from each stratum k. The asymptotic representation of the IPW estimator in Equation (3) then becomes ${\hat{\beta }}_{\text{IPW}}\approx \beta +\frac{1}{N}\sum_{k=1}^{K}\sum_{i\in I_{k}}R_{i}\frac{N_{k}}{n_{k}}h_{i},$ where $I_{k}$ is the set of indices in stratum k so that $\pi_{i}=n_{k}/N_{k}$ for $i\in I_{k}$. Applying the classic optimal allocation result of Neyman [15] in this setting shows that the variance of this estimator for a sample of size n is minimized when

(4) $$n_{k}=n\frac{N_{k}\sigma_{k}}{\sum_{k=1}^{K}N_{k}\sigma_{k}},$$

where $\sigma_{k}$ is the stratum‐specific population standard deviation of $h_{i}$ [20, 37]. Although $\sigma_{k}$ is typically unknown in practice, it can often be approximated using previously collected data. One drawback of Neyman allocation is that the resulting stratum sample sizes are not whole numbers, and they may not add up to the exact total sample size after rounding. In addition, the optimal stratum sizes $n_{k}$ are not constrained to be less than $N_{k}$. Wright [16, 38] presented an exactly optimal solution to this problem by decomposing the variance of the final stratified estimator into a difference of discrete terms. By selecting samples from strata corresponding to the largest of these terms, he derived an integer‐valued algorithm which is hereafter referred to as Neyman–Wright allocation.

### 2.3 Optimal Designs for Generalized Raking

Chen and Lumley [18] derive the optimal design for GR estimators by noting their asymptotic equivalence to generalized regression (GREG) estimators. Let $T_{h}=\sum_{i=1}^{N}h_{i}$ be the total of influence functions among all N units in Phase 1. Then the GREG estimator for $T_{h}$ under stratified sampling is

(5) $${\hat{T}}_{h,GREG}=\overset{K}{\underset{k=1}{\sum }}\underset{i\in I_{k}}{\sum }R_{i}\frac{N_{k}}{n_{k}}(h_{i}-\alpha -\theta^{⊤}c_{i})+\overset{N}{\underset{i=1}{\sum }}(\alpha +\theta^{⊤}c_{i}),$$

where θ is the parameter from a least squares regression of h on c with an intercept α. By the GR analog of Equation (3), minimizing the variance of ${\hat{T}}_{h}$ is asymptotically equivalent to minimizing the variance of the estimator $\hat{\beta }$. If the estimators for α and θ converge at a $\sqrt{n}$ rate, then the variance of the second summation is of smaller order than the variance of the first summation. It thus suffices to minimize the variance of the first summation. The first term is the Horvitz–Thompson IPW estimator of the population total of residuals from the least squares regression of h on c, so, by the arguments of Section 2.2, the optimal design for GR estimators is given by the Neyman allocation in Equation (4), but with $\sigma_{k}$ replaced by the stratum‐specific standard deviation of these residuals.

Despite these differences in the optimal designs for raking and IPW estimators, Chen and Lumley [18] found that they often lead to estimators with similar efficiency. Moreover, their optimal design for GR estimators involves both $h_{i}$ and $c_{i}$, which are not jointly available a priori. Consequently, their results suggest that optimizing sampling for an IPW estimator is reasonable even when using a GR estimator. However, we will later demonstrate that this reasoning does not necessarily extend to the multiple‐parameter setting.

### 2.4 Multiwave Sampling in Two‐Phase Designs

Neyman allocation is particularly useful in two‐phase settings where a strong relationship exists between the Phase 1 data and validated data. In these cases, $\sigma_{k}$ in Equation (4) can be closely approximated by fitting the target regression model on the Phase 1 data to generate estimates $h_{i}^{*}$ of $h_{i}$ and using the within‐stratum standard deviations of $h_{i}^{*}$ as estimates for $\sigma_{k}$ [17, 33]. Further, these $h_{i}^{*}$ can serve as informative components of $c_{i}$ for GR estimation. In practice, however, Phase 1 variables will not always be highly correlated with the true optimal allocation parameters. To handle such scenarios, McIsaac and Cook [19] suggest dividing Phase 2 into a series of subphases, which are often referred to as waves. Information about Phase 2 variables collected in previous waves can then be used to inform sample allocation in subsequent waves. A further benefit of this adaptive, multiwave approach is that it allows strata to be subdivided during Phase 2, thus offering another avenue for efficiency gain. Optimal stratification occurs when optimal allocation assigns an equal number of samples to each stratum [36, 39]. Theoretically, one wave per sampling unit would ensure the maximum amount of information is available at each decision point, but this is rarely practical. Instead, a more modest choice of 2‐6 waves has been found to be useful [8]. The simulations in this work are carried out over 4 waves.

## 3 Considerations for Multiple Parameters

### 3.1 Setup

We now consider the case where the parameter of interest is a vector $\beta ={\left(\beta_{1},\ldots ,\beta_{P}\right)}^{T}$. While replacing the scalars of Section 2 with vectors provides a natural framework for this setting, extending adaptive two‐phase designs to multiple parameters raises several practical considerations regarding allocation criteria, weighting, standardization, and implementation that we address in this section. Following a standard two‐phase sampling setting, we assume that inexpensive variables have been collected for a Phase 1 cohort of size N, but identification of β requires expensive variables that can only be collected for a fixed n<N units in Phase 2. We again assume that an asymptotically linear estimator $\tilde{\beta }$ for β would be available if the Phase 2 variables were observed for the entire cohort, as is the case in standard regression settings. Our aim is to determine a stratified sampling design for selecting the n Phase 2 samples that will lead to an efficient design‐based estimator $\hat{\beta }$ for β.

Although estimation theory extends naturally to this multiple‐parameter setting through vector‐valued influence functions, the optimal design problem is more complicated. The Phase 2 sampling design is shared across all parameters, and the allocation that minimizes the variance of an estimator for one parameter will generally differ from the optimal allocation for another. This setting therefore requires an optimality criterion for balancing efficiency across components of β. In this work, we focus on A‐optimality, which defines the optimal design as the one minimizing a weighted sum of component‐wise variances of an estimator $\hat{\beta }$ for β. While other criteria, such as minimaxity, have been used in similar settings, A‐optimality is interpretable, naturally accommodates priority weighting across parameters, and is well established in the optimal design literature [23, 25, 40].

Write $h_{i}$ for the P‐dimensional influence function vector corresponding to the estimator $\tilde{\beta }$ of β for person i. By Equation (2), an estimator for β if the Phase 2 variables were observed for the entire Phase 1 cohort can, for large N, be well approximated by $\tilde{\beta }=\beta +\frac{1}{N}\sum_{i=1}^{N}h_{i}$. However, these variables, and hence this total, are unknown. Instead, we estimate the sum by conducting stratified random sampling to select $n_{k}$ samples from the $N_{k}$ in each stratum.

For stratum k, we define the within‐stratum covariance matrix of $h_{i}$ as $V_{k}$, a P×P matrix with (j,l)‐th entry $\sigma_{jl,k}\equiv {\text{cov}}_{k}(h_{j},h_{l})$. When j=l, we write $\sigma_{jl,k}=\sigma_{j,k}^{2}={\mathrm{Var}}_{k}(h_{j})$. Letting $w_{k}=\frac{N_{k}}{n_{k}}$, we have the IPW estimator ${\hat{\beta }}_{\text{IPW}}\approx \beta +\frac{1}{N}\sum_{k}\sum_{i\in I_{k}}R_{i}w_{k}h_{i}$ and

(6) $$\mathrm{Var}({\hat{\beta }}_{\text{IPW}}|\text{phase 1})=\overset{K}{\underset{k=1}{\sum }}\frac{N_{k}^{2}}{n_{k}}V_{k}-\overset{K}{\underset{k=1}{\sum }}N_{k}V_{k}.$$

The second term, which reflects the finite population correction, only depends on the fixed $N_{k}$ and $V_{k}$ and not on the Phase 2 allocation. Thus, minimizing the variance with respect to the Phase 2 design is equivalent to selecting the $n_{k}$'s that minimize the first term subject to $\sum_{k=1}^{K}n_{k}=n$.

### 3.2 Simple Strategies: Independent Allocation for Each Parameter

A natural approach to optimize allocation for multiple parameters in a single study is to divide the budget of n Phase 2 sampling units into P pieces, one for each parameter of interest, and then determine the allocation for each group independently using known Neyman or Neyman–Wright allocation results for the IPW estimator. Let $n^{\left[p\right]}$ be the number of Phase 2 samples to be allocated optimally with respect to the p‐th parameter of interest, where $\sum_{p=1}^{P}n^{\left[p\right]}=n$. Also, let $n_{k}^{\left[p\right]}$ denote the sample size for stratum k with $\sum_{k=1}^{K}n_{k}^{\left[p\right]}=n^{\left[p\right]}$. The overall optimal allocation for $n_{k}$ becomes

(7) $$n_{k}=\overset{P}{\underset{p=1}{\sum }}n_{k}^{\left[p\right]}=\overset{P}{\underset{p=1}{\sum }}n^{\left[p\right]}\frac{N_{k}\sigma_{p,k}}{\sum_{k^{\prime }=1}^{K}N_{k^{\prime }}\sigma_{p,k^{\prime }}},$$

where $\sigma_{p,k}$ is the square root of the p‐th diagonal entry of $V_{k}$. This is equivalent to the sum of the individual Neyman allocations for each parameter of interest. Alternatively, one could derive and sum across the Wright [16] integer‐valued optimal design.

If Phase 2 is conducted over multiple waves, the above allocation may be implemented in multiple ways. Let $n_{k}^{\left(t\right)}$ be the sample size in stratum k at wave t=1,…,T and $n^{\left(t\right)}=\sum_{k=1}^{K}n_{k}^{\left(t\right)}$. Two possible approaches are:

- Simultaneous approach: At each wave t, recompute the cumulative optimal allocation for $\sum_{v=1}^{t}n^{\left(v\right)}$ samples according to Equation (7), or the Neyman–Wright equivalent, and set $n_{k}^{\left(t\right)}$ to be the difference between the cumulative allocation and the units already sampled. Sampling at each wave is conducted with respect to all outcomes of interest, and allocation for both outcomes can make equal use of all information from prior waves.
- Sequential approach: At each wave, calculate the cumulative optimal allocation for $\sum_{v=1}^{t}n^{\left(v\right)}$ samples with respect to a single outcome using Equation (4) or the Neyman–Wright equivalent, changing the parameter that determines the allocation across waves and again setting $n_{k}^{\left(t\right)}$ to be the difference between the cumulative allocation and the units already sampled. This is the approach used by Shepherd et al. [8]. One benefit of this approach is that different strata can be defined for each outcome (See Figure 1).

**FIGURE 1:**
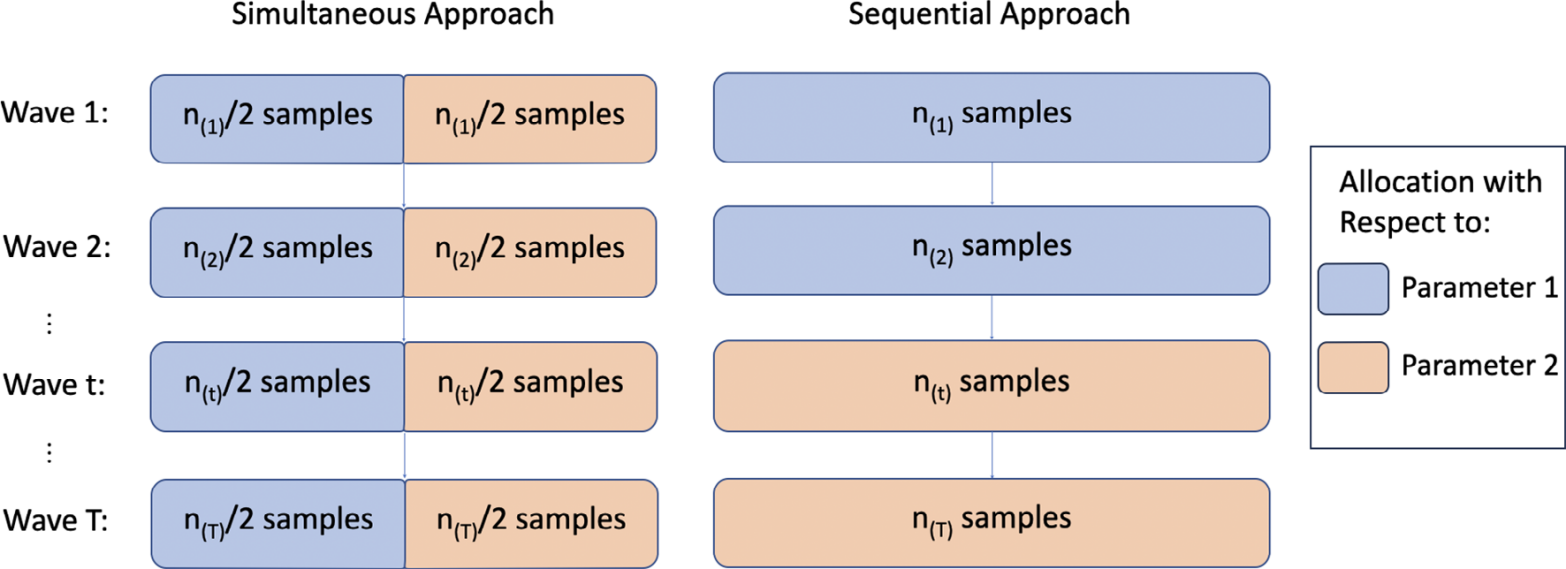
Visual depiction of simultaneous and sequential approaches when two parameters are of equal interest.

### 3.3 Prioritized A‐Optimality

The A‐optimality approach is to minimize the sum of the variances for the p parameter estimates [41]. That is, to minimize $\text{trace}\left(\mathrm{Var}(\hat{\beta })\right)=\sum_{p=1}^{P}\sum_{k=1}^{K}\frac{N_{k}^{2}}{n_{k}}\sigma_{p,k}^{2}$ in the IPW case. Because the parameters may have varying levels of importance towards the research goals, it may be useful to allow this sum to be weighted. Denote $a_{p}$ as the predefined priority weight for the pth parameter estimate, where $\sum_{p=1}^{P}a_{p}=1$ for convenience. Traditional (nonprioritized) A‐optimality now becomes a special case where all weights are equal. We want to find

(8) $${\text{argmin}}_{n_{1},\ldots ,n_{K}}\overset{P}{\underset{p=1}{\sum }}\left[a_{p}\overset{K}{\underset{k=1}{\sum }}\frac{N_{k}^{2}}{n_{k}}\sigma_{p,k}^{2}\right]=\underset{n_{1},\ldots ,n_{K}}{\text{argmin}}\;\overset{K}{\underset{k=1}{\sum }}\frac{N_{k}^{2}\sum_{p=1}^{P}a_{p}\sigma_{p,k}^{2}}{n_{k}},$$

subject to the constraint $\sum_{k=1}^{K}n_{k}=n$. The continuous solution can be obtained using Lagrange multipliers (see Rivera‐Rodriguez et al. [22]) as

$$n_{k}=n\frac{N_{k}\sqrt{\sum_{p=1}^{P}a_{p}\sigma_{p,k}^{2}}}{\sum_{k=1}^{K}N_{k}\sqrt{\sum_{p=1}^{P}a_{p}\sigma_{p,k}^{2}}},$$

which is the Neyman allocation with the standard deviation of a single variable replaced by $\sqrt{\sum_{p=1}^{P}a_{p}\sigma_{p,k}^{2}}$.

We can also extend the Neyman–Wright algorithm to obtain an exact, integer‐valued solution to Equation (8) satisfying $n_{k}\geq 2$ (for unbiased variance estimation) and $n_{k}<N_{k}$. This allocation, whose derivation is provided in Supplemental Section S1, is given by forming the array of priority values

(9) $$\begin{array}{ll} & \frac{N_{1}\sqrt{\sum_{p=1}^{P}a_{p}\sigma_{p,1}^{2}}}{\sqrt{2\cdot 3}}\;\frac{N_{1}\sqrt{\sum_{p=1}^{P}a_{p}\sigma_{p,1}^{2}}}{\sqrt{3\cdot 4}}\\ & \frac{N_{1}\sqrt{\sum_{p=1}^{P}a_{p}\sigma_{p,1}^{2}}}{\sqrt{4\cdot 5}}\;\cdots \\ & \;\vdots \\ & \frac{N_{k}\sqrt{\sum_{p=1}^{P}a_{p}\sigma_{p,k}^{2}}}{\sqrt{2\cdot 3}}\;\frac{N_{k}\sqrt{\sum_{p=1}^{P}a_{p}\sigma_{p,k}^{2}}}{\sqrt{3\cdot 4}}\\ & \frac{N_{k}\sqrt{\sum_{p=1}^{P}a_{p}\sigma_{p,k}^{2}}}{\sqrt{4\cdot 5}}\;\cdots \\ & \;\vdots \\ & \frac{N_{K}\sqrt{\sum_{p=1}^{P}a_{p}\sigma_{p,K}^{2}}}{\sqrt{2\cdot 3}}\;\frac{N_{K}\sqrt{\sum_{p=1}^{P}a_{p}\sigma_{p,K}^{2}}}{\sqrt{3\cdot 4}}\\ & \frac{N_{K}\sqrt{\sum_{p=1}^{P}a_{p}\sigma_{p,K}^{2}}}{\sqrt{4\cdot 5}}\;\cdots \end{array}$$

and taking the number of samples assigned to stratum k as 2 plus the number of priority values in the k‐th row that are among the largest n−2K in the entire array. In the context of multiple parameters, where the number of strata is typically large in order to accommodate the different parameters and some strata are hence small, this algorithm proves especially useful because, unlike Neyman allocation, it incorporates the constraint $n_{k}<N_{k}$.

### 3.4 Optimal Design for Generalized Raking With Multiple Parameters

The approaches detailed so far for IPW estimators extend naturally to GR estimators by the arguments of Section 2.3. Let $\epsilon_{i,p}$ be the residual from the least squares regression of $h_{i,p}$ on the raking variables $c_{i,p}$. In practice, these quantities are unknown but can be estimated using available data. The influence functions $h_{i,p}$ can be estimated using the validated Phase 2 data, while the raking variables $c_{i,p}$ may be constructed from the Phase 1 influence function proxies or estimated as approximations to the optimal raking variables $c_{i,p}^{\text{opt}}$ using regression or imputation. Denote by $\sigma_{p,k}^{\left(\epsilon \right)}$ the standard deviation of $\epsilon_{i,p}$ within stratum k. Notably, $\sigma_{p,k}^{\left(\epsilon \right)}$ reflects within‐stratum variation unexplained by the raking variables, while $\sigma_{p,k}$ reflects overall within‐stratum variation. The independent allocation and prioritized A‐optimality strategies for GR estimators are given by replacing $\sigma_{p,k}$ with $\sigma_{p,k}^{\left(\epsilon \right)}$ in Equations (7) and (9), respectively.

Although Chen and Lumley [18] showed that the optimal designs for IPW and GR often lead to similar efficiencies for a single parameter, this formulation suggests that the multiple‐parameter setting is more complicated. In the single‐parameter setting, a poor raking variable that is weakly correlated with $h_{i}$ will lead to θ≈0 in Equation (5), making the GR and IPW estimators and optimal designs effectively equivalent. On the other hand, a strong raking variable will lead to $h_{i}-\alpha -\theta^{⊤}c_{i}\approx 0$ in Equation (5), meaning the variance contributed by Phase 2 sampling will be minimal even for suboptimal designs. Hence, the GR optimal design will only lead to efficiency gains over the IPW design when the variances of residualized influence functions differ notably across strata (e.g., differential measurement error). In the multiple‐parameters setting, differences in the level of correlation between $h_{i,p}$ and their raking variables in $c_{i,p}$ across parameters p will also result in a meaningful advantage for the GR A‐optimal design. For instance, if $c_{i,p}$ contains a variable that is highly correlated with $h_{i,p}$ but $h_{i,p^{\prime }}$ is weakly correlated with each component of $c_{i,p^{\prime }}$, then the GR A‐optimal design will closely resemble the IPW optimal design for ${\hat{\beta }}_{p^{\prime }}$. The IPW A‐optimal design, however, would “waste” $a_{p}$ of the sampling efforts trying to optimize for ${\hat{\beta }}_{p}$, even though GR will lead to a small variance regardless of its design. In the measurement error setting, this occurs when the variables relating to one parameter are measured with high error, but for another parameter they are measured with low error. Such a phenomenon is common in EHR‐based studies, and it is highlighted in the data example in Section 5.

### 3.5 Practical Implementation

Algorithm 1 describes the specific steps to implement a multiwave, prioritized A‐optimal design. In Supplemental Section S1.2, we establish that this adaptive design converges asymptotically to the oracle prioritized A‐optimal design. The algorithm leaves several implementation choices unspecified, including the number and size of the waves and the method used to estimate the raking variables. Appropriate choices depend on the total Phase 2 sample size, the quality of the Phase 1 variables, and other practical considerations. For instance, imputation‐based approximations to the optimal raking variables may be particularly useful when the Phase 1 variables are highly error‐prone or the error structure is complex, while simpler approaches such as influence function proxies constructed from Phase 1 data alone may perform similarly in other settings [11, 35]. Notably, the latter approach only requires Phase 1 data, while the former may also use Phase 2 data. When Phase 2 data are used to estimate the raking variables, separate data folds should be used to estimate the raking variables and influence functions when calculating the residuals for the GR‐optimal design to avoid underestimating residual variation, as described by Chen and Lumley [18].

A key feature of Algorithm 1 is that, at each wave t, it targets the oracle allocation that would be optimal for $\sum_{s=1}^{t}n^{\left(s\right)}$ samples. It then tries to achieve this optimal design in line 13 by allocating the available $n^{\left(t\right)}$ to fill the slots that have not already been sampled in the first t−1 waves of sampling. A complication arises when the estimated allocation assigns fewer observations to a stratum than have already been sampled in previous waves. Yang et al. [42] refer to this phenomenon as oversampling and discuss several strategies with available software for addressing it. In practice, oversampling is typically minor and is often corrected in subsequent waves. Moreover, its impact is asymptotically negligible when the number of waves increases with the Phase 2 sample size.

As with other adaptive multiwave procedures, the quality of the proposed algorithm depends on the accuracy of the estimated nuisance parameters, which in this case are the within‐stratum residual variances. These estimates become more reliable as Phase 2 data accrue but may be unstable in smaller studies or when few Phase 2 observations are available in some strata. Prior to wave 1, there are no Phase 2 data available to estimate the GR‐optimal design, so the algorithm proposes targeting the optimal IPW design. Case‐control sampling, simple random sampling, proportional sampling, or a prior‐based allocation for the wave 1 design are reasonable alternatives, which may also help with the stability of nuisance parameter estimates for later waves [37]. In settings where stability could be a concern even in later waves, improved finite‐sample performance may be achieved by using a larger initial wave, fewer waves, or regularized estimation of the raking variables and residual variances.

A crucial input to the algorithm is the vector of priority weights $a_{1},...,a_{p}$. When no parameter is of special interest, equal weights provide a natural default. In other settings where a subset of the p parameters are of heightened interest, assigning them larger priority weights will lead to a design that targets their precise estimation. Sensitivity analyses across several plausible choices of priority weights can also be used to assess how strongly the optimal allocation depends on this input. Because the prioritized A‐optimal criterion is sensitive to parameter scale, parameters measured on different scales should generally be standardized before computing the allocation. This may be accomplished by standardizing the variables before entering them into the optimal design formula, or by dividing each parameter's contribution to the allocation criterion by the overall variance of its influence function. Since these variances are generally unknown, they may be estimated from the Phase 1 influence‐function proxies and updated as Phase 2 data accumulate.

ALGORITHM 1 **Adaptive multiwave A‐optimal sampling design for generalized raking.**

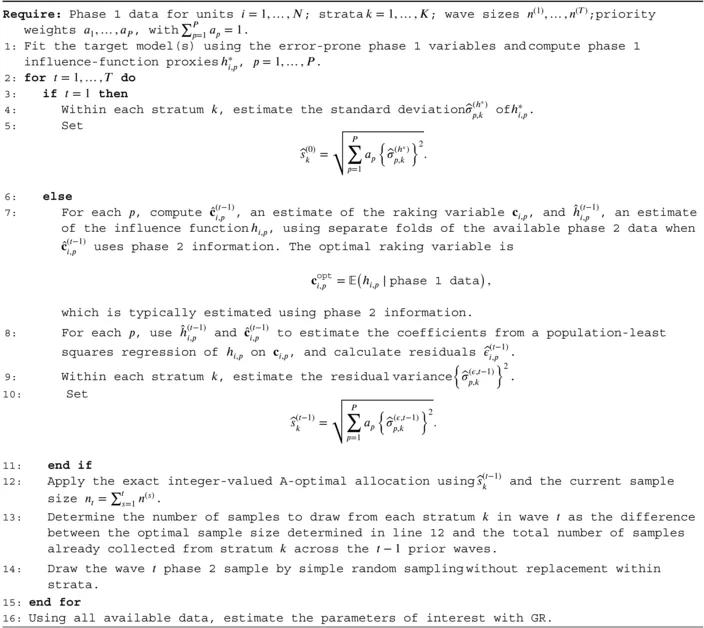

## 4 Simulation Study

We conducted a series of simulations to compare the performance of different allocation strategies in the context of two‐phase, multiwave sampling with multiple parameters of interest. We considered the problem of efficiently estimating logistic regression coefficients from the following models:

(10) $$\begin{array}{ll} Pr\left(Y_{1i}|X_{1i},X_{2i},Z_{i},Y_{2i}\right) & =\text{expit}(\beta_{01}+\beta_{11}X_{1i}+\beta_{21}X_{2i}\\ & \;+\beta_{31}Z_{i}+\beta_{41}Y_{2i})\\ Pr\left(Y_{2i}|X_{1i},X_{2i},Z_{i}\right) & =\text{expit}(\beta_{02}+\beta_{12}X_{1i}+\beta_{22}X_{2i}+\beta_{32}Z_{i}). \end{array}$$

Motivated by real‐world examples, we focused on the measurement error setting, which can be viewed as a special case of missing data in which the error‐free Phase 2 variables are missing at Phase 1, but error‐prone Phase 1 variables are correlated with the missing values. Within this framework, we considered scenarios corresponding to different parameters of interest and features of the data‐generating process, including correlation levels and the degree of measurement error in Phase 1 variables (Table 1). These scenarios fall into three broad categories: two outcomes of interest (Scenario 2O), two predictors of interest (Scenario 2P), and two outcomes and two predictors of interest (Scenario 2O2P).

**TABLE 1:** Simulation scenarios.

Scenario		Coefficients of interest	Cor($Y_{1},Y_{2}$)	Cor($X_{1},X_{2}$)	Error level
Setting	Case				
Two Outcomes, One Predictor (2O)	A	$\beta_{11},\beta_{12}$	Low	—	Low
	B	$\beta_{11},\beta_{12}$	Low	—	High
	C	$\beta_{11},\beta_{12}$	High	—	Low
	D	$\beta_{11},\beta_{12}$	High	—	High
One Outcome, Two Predictors (2P)	A	$\beta_{12},\beta_{22}$	—	Low	Low
	B	$\beta_{12},\beta_{22}$	—	Low	High
	C	$\beta_{12},\beta_{22}$	—	High	Low
	D	$\beta_{12},\beta_{22}$	—	High	High
Two Predictors, Two Outcomes (2O2P)	A	$\beta_{11},\beta_{21},\beta_{12},\beta_{22}$	Moderate	Moderate	Low
	B	$\beta_{11},\beta_{21},\beta_{12},\beta_{22}$	Moderate	Moderate	High

Our two motivating examples fall under Scenario 2O, but can easily be extended to the others. The first is the VCCC HIV/AIDS cohort, where the relationship between CD4 cell count and two binary outcomes, AIDS‐defining event (ADE) and death, is of interest [7, 43]. These two outcomes are highly correlated because AIDS may lead to death, and similar causes may also lead to both AIDS and death independently. A second motivating example is the Vanderbilt Maternal Weight Study, where the relationship between maternal weight change during pregnancy and two outcomes with low correlation, childhood obesity and asthma, was of interest [8]. In both studies, Phase 1 consisted of error‐prone measurements obtained from electronic health records, while Phase 2 involved chart review to obtain error‐free measurements for the variables of interest.

### 4.1 Setup

At each simulation iteration, we generated a Phase 1 dataset with *N* = 10 000 samples including two continuous variables, $X_{1}$ and $X_{2}$, one binary covariate Z, and two binary outcome variables $Y_{1}$ and $Y_{2}$ generated according to the logistic models in (10). We also simulated error‐prone or proxy versions of each variable, denoted $X_{1}^{*},X_{2}^{*},Z^{*},Y_{1}^{*},Y_{2}^{*}$, and assumed only these versions were collected in Phase 1. The measurement errors were simulated with correlation structures to reflect realistic error structures. We considered varying degrees of measurement error for the outcome and exposure(s) of interest. Further details on the simulation setup are provided in Supplemental Section S2.

The key distinguishing features between scenarios are the parameters of interest and the level of correlation between $Y_{1}$ and $Y_{2}$ or between $X_{1}$ and $X_{2}$. Scenarios 2O‐A and 2O‐B reflect the case of nearly independent outcomes, as in the Vanderbilt Maternal Weight Study, and Scenarios 2O‐C and 2O‐D reflect the case of correlated outcomes, as in the VCCC study, where one is a predictor for the other. Across scenarios, the prevalences of $Y_{1}$ and $Y_{2}$ were maintained at approximately 0.2 and 0.4, respectively, representing rare and common outcomes. Further, all continuous covariates were generated with variance 1 to minimize the contribution of scale. In practice, this would involve standardizing the covariates. For the primary analyses, we assume that the variances of the coefficients of interest are of equal importance, that is, the weights $a_{p}$ in Equation (8) are all equal. We additionally explore the sensitivity of results for Scenario 2O‐A across different choices of A‐optimality priority weights.

### 4.2 Sampling Designs

For each of the ten combinations of correlation and measurement error levels, five Phase 2 sampling design strategies were implemented assuming a Phase 2 budget of n=1000 validation samples:

- *Strategy 1*: Case‐control sampling in a single wave.
- *Strategy 2*: Simultaneous independent allocation for each parameter in four equally sized waves. At each wave, $n_{\left(t\right)}=250$ samples were split evenly among the parameters of interest, with each subset allocated optimally for a single parameter (e.g., in the two‐parameter case, 125 samples were optimized independently for each parameter).
- *Strategy 3*: Sequential independent allocation for each parameter over four equally sized waves. At each wave, $n_{\left(t\right)}=250$ samples were allocated optimally for a single parameter, with each parameter receiving an equal number of dedicated waves.
- *Strategy 4*: Strategy 3, but with the order of parameter‐specific optimization reversed.
- *Strategy 5*: Prioritized A‐optimality over four equally sized waves.

Further details on each strategy are provided in Supplemental Section S2.3. We also consider a supplemental setting where n=500.

Estimation of the final regression parameters of interest was performed using both GR and IPW. For strategies 2‐5, strata were formed based on combinations of $Y_{1}^{*},Y_{2}^{*}$ and categorized versions of $X_{1}^{*}$ and $X_{2}^{*}$ defined by quantile cut points. These stratum boundaries were motivated by Amorim et al. [39] and Särndal et al. [36], who note that efficient stratification yields approximately equal sample allocations across strata under Neyman allocation, and they were held fixed throughout Phase 2 sampling for simplicity. Further details on the scenario‐specific stratum constructions are provided in Supplemental Section S2.3.

Optimum allocation for wave 1 was calculated using Neyman allocation of the estimated influence functions $h_{i}^{*}$ from fitting the target model on the error‐prone Phase 1 data for both IPW and GR estimators. For subsequent waves under the IPW estimator, IPW‐optimal Neyman allocation was performed using influence functions from the target model fit to the previously collected Phase 2 data with IPW estimators. For GR estimators, the GR‐optimal approach detailed in Section 3.4 and Algorithm 1 was used, with the estimated influence functions from fitting the target model on the Phase 1 data as the auxiliary raking variables (see Supplemental Section S2.4 for details). The R packages ‘survey’ and ‘optimall’ were used to implement the estimation and design procedures, respectively [30, 42]. Empirical variances over 2500 simulation iterations were reported as the primary results. Estimated 95% confidence intervals and their empirical coverage probabilities were also reported as secondary results, with details of the methods used for variance estimation described in Supplemental Section S2.5.

### 4.3 Simulation Results

The empirical variances of the regression‐coefficient estimates for each estimator for simulation Scenarios 2O, 2P, and 2O2P are presented in Tables 2 and 3, and Supplemental Table S3, respectively. Across all scenarios, GR estimators outperformed IPW estimators for each sampling strategy, with the largest efficiency gain from raking observed under the nonoptimal case‐control design. In general, the efficiency improvement from raking relative to IPW under case‐control sampling was comparable in magnitude to that achieved by A‐optimal sampling with IPW estimators relative to case‐control sampling, and combining the two provided an additional but more modest gain. Supplemental Tables S4–S6 report empirical mean squared errors (MSEs), which followed similar patterns to the empirical variances, and Supplemental Tables S8 and S9 show that the empirical coverage probabilities of estimated 95% confidence intervals are close to the nominal 0.95 level across strategies. The trends across allocation strategies were consistent for both IPW and GR estimators, and results in this section focus on the latter unless noted otherwise.

**TABLE 2:** Empirical variance ($\times 10^{3}$) for each strategy under Scenario 2O: two outcomes of interest.

Corr. level	Error level	Estimator	Parameter	Strat 1: Case‐control	Strat 2: Simultaneous	Strat 3: $\beta_{12}$ last	Strat 4: $\beta_{11}$ last	Strat 5: A‐optimal
Low	Low (A)	IPW	Var(${\hat{\beta }}_{11}$)	5.58	2.54	3.05	2.56	2.71
			Var(${\hat{\beta }}_{12}$)	6.17	2.71	2.41	3.18	2.53
		GR	Var(${\hat{\beta }}_{11}$)	2.23	1.93	2.13	1.88	1.93
			Var(${\hat{\beta }}_{12}$)	2.61	2.09	1.93	2.49	2.13
	High (B)	IPW	Var(${\hat{\beta }}_{11}$)	5.75	4.97	5.52	4.52	4.82
			Var(${\hat{\beta }}_{12}$)	5.30	3.83	3.69	4.23	3.62
		GR	Var(${\hat{\beta }}_{11}$)	4.55	4.44	5.08	4.37	4.56
			Var(${\hat{\beta }}_{12}$)	4.05	3.53	3.36	3.93	3.60
High	Low (C)	IPW	Var(${\hat{\beta }}_{11}$)	7.44	4.25	4.46	4.06	4.11
			Var(${\hat{\beta }}_{12}$)	7.47	3.36	3.30	4.28	3.42
		GR	Var(${\hat{\beta }}_{11}$)	3.55	2.96	3.10	2.88	2.87
			Var(${\hat{\beta }}_{12}$)	3.35	2.66	2.39	2.97	2.66
	High (D)	IPW	Var(${\hat{\beta }}_{11}$)	8.64	7.55	7.96	7.42	7.46
			Var(${\hat{\beta }}_{12}$)	6.76	4.98	4.54	5.41	4.86
		GR	Var(${\hat{\beta }}_{11}$)	7.12	7.01	7.55	6.62	6.49
			Var(${\hat{\beta }}_{12}$)	5.04	4.58	4.17	5.17	4.40

**TABLE 3:** Empirical variance ($\times 10^{3}$) for each strategy under Scenario 2P: two predictors of interest.

Corr. level	Errorlevel	Estimator	Parameter	Strat 1: Case‐control	Strat 2: Simultaneous	Strat 3: $\beta_{22}$ last	Strat 4:$\beta_{12}$ last	Strat 5: A‐optimal
Low	Low (A)	IPW	$\mathrm{Var}({\hat{\beta }}_{12})$	6.38	4.67	4.83	4.68	4.65
			$\mathrm{Var}({\hat{\beta }}_{22})$	8.16	6.03	5.69	5.97	5.83
		GR	$\mathrm{Var}({\hat{\beta }}_{12})$	3.91	3.65	3.52	3.77	3.58
			$\mathrm{Var}({\hat{\beta }}_{22})$	5.81	5.11	5.04	5.25	5.01
	High (B)	IPW	$\mathrm{Var}({\hat{\beta }}_{12})$	6.94	5.93	5.65	6.03	5.92
			$\mathrm{Var}({\hat{\beta }}_{22})$	8.43	6.88	6.81	6.90	7.24
		GR	$\mathrm{Var}({\hat{\beta }}_{12})$	5.58	5.15	5.10	5.34	5.19
			$\mathrm{Var}({\hat{\beta }}_{22})$	6.98	6.82	7.01	6.55	6.19
High	Low (C)	IPW	$\mathrm{Var}({\hat{\beta }}_{12})$	11.14	8.56	8.90	9.05	9.00
			$\mathrm{Var}({\hat{\beta }}_{22})$	12.60	9.67	10.26	9.95	10.00
		GR	$\mathrm{Var}({\hat{\beta }}_{12})$	7.74	7.17	6.95	6.92	6.87
			$\mathrm{Var}({\hat{\beta }}_{22})$	10.02	9.11	8.76	8.41	8.47
	High (D)	IPW	$\mathrm{Var}({\hat{\beta }}_{12})$	11.49	10.42	10.88	10.91	10.64
			$\mathrm{Var}({\hat{\beta }}_{22})$	13.55	11.72	12.50	11.84	11.51
		GR	$\mathrm{Var}({\hat{\beta }}_{12})$	9.35	9.58	9.49	9.62	9.68
			$\mathrm{Var}({\hat{\beta }}_{22})$	11.52	11.15	11.21	10.99	10.83

Across all ten simulation scenarios, multiwave adaptive sampling with Neyman allocation of influence functions (Strategies 2‐5) yielded more efficient regression‐coefficient estimates than case‐control sampling (Strategy 1). Table 4 reports the empirical relative efficiency (ERE) of the GR estimators for each strategy, where ERE is defined as the ratio of the total empirical variance of the estimated regression coefficients under the A‐optimal design to that under the given design. A‐optimal designs (Strategy 5) achieved the best or nearly the best empirical relative efficiency in every scenario. The simultaneous and sequential allocation strategies (Strategies 2‐4) generally yielded variances close to those of the A‐optimal design, though they typically performed slightly worse.

**TABLE 4:** Empirical relative efficiency for the sum of variances for each strategy under GR.

Scenario		Corr. level	Errorlevel	Strat 1: Case‐control	Strat 2: Simultaneous	Strat 3: Sequentialorder 1	Strat 4: Sequential order 2	Strat 5: A‐optimal
Setting	Case							
2O	A	Low	Low	0.84	1.01	1.00	0.93	1.00
	B	Low	High	0.95	1.02	0.97	0.98	1.00
	C	High	Low	0.80	0.98	1.01	0.94	1.00
	D	High	High	0.90	0.94	0.93	0.92	1.00
2P	A	Low	Low	0.88	0.98	1.00	0.95	1.00
	B	Low	High	0.91	0.95	0.94	0.96	1.00
	C	High	Low	0.86	0.94	0.98	1.00	1.00
	D	High	High	0.98	0.99	0.99	1.00	1.00
2O2P	A	Moderate	Low	0.84	0.99	0.98	—	1.00
	B	Moderate	High	0.95	1.01	0.98	—	1.00

*Note:* Scenario 2O: two outcomes, one predictor of interest; Scenario 2P: one outcome, two predictors of interest; Scenario 2O2P: two outcomes, two predictors of interest.

In Scenario 2O, the sequential allocation strategies (Strategies 3 and 4) often minimized variance for the coefficient optimized in the final two waves at the expense of efficiency for the coefficient optimized in the first two waves. For instance, Strategy 3, which optimized for $\beta_{12}$ in the last two waves of Phase 2, was the most efficient design for $\beta_{12}$ but the least efficient (excluding case‐control) for $\beta_{11}$ in all four sub‐scenarios (Table 2). Similarly, Strategy 4 was the least efficient approach for $\beta_{12}$ in all four sub‐scenarios but the most efficient for $\beta_{11}$ in two. In Scenario 2O2P, the sequential approach (Strategy 3) led to the lowest ERE at both error levels.

In the analysis examining the effect of varying priority weights for the A‐optimal strategy in Scenario 2O‐A, increasing the priority weight assigned to $\beta_{11}$ reduced the empirical variance of ${\hat{\beta }}_{11}$ while increasing the variance of ${\hat{\beta }}_{12}$, demonstrating the tradeoff induced by prioritizing one parameter over another (Supplemental Table S10). Equal weighting yielded relatively balanced efficiency across the two parameters, and the most pronounced loss in precision for the less‐prioritized parameter occurred when its weight was set to zero. In contrast, even a 3:1 imbalance in priority weights resulted in only modest increases in variance for the less‐prioritized parameter, suggesting that assigning any nonnegligible priority to a parameter can substantially improve its efficiency relative to zero priority.

Empirical variances increased with higher levels of measurement error across all sampling strategies. Additionally, the efficiency gains from GR compared to IPW diminished in high‐error settings, where the Phase 1 raking variables were less informative. Similarly, the efficiency advantage of optimal allocation over case‐control sampling (Strategy 1) declined as measurement error increased. The largest empirical relative efficiency gain from A‐optimal design in a high‐error scenario was 0.10 in Scenario 2O‐D, while the gains in low‐error scenarios were all larger than 0.10 and as high as 0.20 in Scenario 2O‐C (Table 4). The relative efficiency gains from adaptive, multiwave sampling were fairly consistent across different levels of correlation between the outcomes or predictors of interest. Results for the supplemental scenario with n=500 (Supplemental Tables S11–S13) were broadly consistent with the primary findings.

## 5 Example: Vanderbilt Comprehensive Care Clinic Study

We also assessed the performance of the various optimal allocation strategies using real EHR data collected on 1595 HIV‐positive persons who received care at the VCCC between 1999 and 2013. This dataset served as a motivating example for our work, as both death and ADE have been considered as outcomes of interest in prior studies, particularly as they relate to CD4 count at the time of antiretroviral therapy (ART) initiation [7, 43]. These three variables, along with many others covering demographic information, medical history, and laboratory test results, were collected routinely during patient visits to the clinic and stored in the EHR. Given the error‐prone nature of EHR‐derived data, the research team also conducted validation by chart review for the entire cohort, which revealed many errors in EHR‐derived variables relating to the ADE analysis. Notably, time to ADE was measured incorrectly in the EHR for 34.5% of patients, and 10.8% were found to have an incorrect ADE status (sensitivity: 0.87; specificity: 0.89; positive predictive value: 0.35). In contrast, variables relating to the death analysis were more accurate in the EHR. Time to death was initially measured with error in only 21.3% of patients, and the indicator for death did not have any errors at all. Further details on this data set and discrepancies between routinely‐collected and validated versions can be found in [7].

In this example, we used the validated data on the whole cohort to assess the performance of optimum allocation strategies assuming the validation budget had been limited to 200 individuals. We defined the “true” parameter values for the coefficients of interest, $\beta_{\mathrm{CD}4,\mathrm{death}}$ and $\beta_{\mathrm{CD}4,\mathrm{ADE}}$, as the coefficients obtained by fitting the following Cox regression models on the entire validated data set:

$$\begin{array}{ll} \lambda_{\text{Death}}(t|\mathrm{CD}4,\mathrm{Age}) & =\lambda_{0,\mathrm{Death}}(t)\cdot \exp(\beta_{\mathrm{CD}4,\mathrm{Death}}\cdot \mathrm{CD}4\\ & \;+\beta_{\mathrm{Age},\mathrm{Death}}\cdot \text{Age})\\ \lambda_{\text{ADE}}(t|\mathrm{CD}4,\mathrm{Age}) & =\lambda_{0,\mathrm{ADE}}(t)\cdot \exp(\beta_{\mathrm{CD}4,\mathrm{ADE}}\cdot \mathrm{CD}4\\ & \;+\beta_{\mathrm{Age},\mathrm{ADE}}\cdot \text{Age}). \end{array}$$

Notably, this setup differs from the simulation study in the previous section where the parameters of interest were logistic regression coefficients from the superpopulation from which Phase 1 was a simple random sample. The model difference highlights the flexibility of the proposed approach since estimators for coefficients in both logistic and Cox regression are asymptotically linear [44]. The difference in sampling framework isolates the Phase 2 contribution to the variance, but this is the only component of the variance that can be controlled through the Phase 2 sampling design, so the optimal allocation results apply as before.

We performed Phase 2 sampling across four waves of 50 samples using each optimum allocation strategy and compared the empirical variances observed over 1000 sampling iterations. The procedure was implemented separately under optimal allocation for IPW and for GR, as well as under the suboptimal case‐control design, following the procedures described in Section 4 and Supplemental Section S2.4. For the adaptive sampling strategies, strata were defined by first cross‐classifying individuals by their Phase 1 ADE status and death status into four groups. The group with neither an observed ADE nor death contained approximately 77% of this cohort initially and was therefore further divided into five strata according to quantiles of the error‐prone CD4 measure within that group, yielding eight strata in total. For the case‐control strategy, 50 samples were collected from each of the four ADE‐by‐death groups. For Strategy 5, equal priority weights were used because $\beta_{\mathrm{CD}4,\mathrm{Death}}$ and $\beta_{\mathrm{CD}4,\mathrm{ADE}}$ were considered of equal scientific interest and are on comparable scales. Table 5 displays the results for both IPW and GR estimators under each sampling approach. Comparisons between the IPW and GR estimators under the same sampling design isolate the efficiency gained from raking, while comparisons between the IPW‐optimal and GR‐optimal designs for the same estimator isolate the effect of tailoring the Phase 2 design to the estimator. The four combinations are shown to distinguish these two sources of efficiency.

**TABLE 5:** Empirical variance ($\times 10^{6}$) from Phase 2 of estimators (with type of design optimality) under each strategy in the VCCC data example.

Estimator	Design	Parameter	Strat 1: Case‐control	Strat 2: Simultaneous	Strat 3: ADE first	Strat 4: Death first	Strat 5:A‐optimal
IPW	IPW‐optimal	$\mathrm{Var}({\hat{\beta }}_{\mathrm{CD}4,\text{ADE}})$	3.08	2.16	2.53	2.21	1.97
		$\mathrm{Var}({\hat{\beta }}_{\mathrm{CD}4,\text{Death}})$	0.87	0.64	0.64	0.79	0.80
GR	IPW‐optimal	$\mathrm{Var}({\hat{\beta }}_{\mathrm{CD}4,\text{ADE}})$	2.62	1.99	2.28	2.07	1.74
		$\mathrm{Var}({\hat{\beta }}_{\mathrm{CD}4,\text{Death}})$	0.03	0.05	0.05	0.06	0.06
IPW	GR‐optimal	$\mathrm{Var}({\hat{\beta }}_{\mathrm{CD}4,\text{ADE}})$	3.08	1.87	2.18	1.77	1.59
		$\mathrm{Var}({\hat{\beta }}_{\mathrm{CD}4,\text{Death}})$	0.87	0.85	0.91	0.95	1.37
GR	GR‐optimal	$\mathrm{Var}({\hat{\beta }}_{\mathrm{CD}4,\text{ADE}})$	2.62	1.65	1.98	1.43	1.24
		$\mathrm{Var}({\hat{\beta }}_{\mathrm{CD}4,\text{Death}})$	0.03	0.04	0.03	0.05	0.05

### 5.1 Example: Results

The data example results further demonstrate the utility of adaptive, multiwave sampling with GR on a real‐world dataset. A‐optimality targeted to the appropriate IPW or GR estimator successfully minimized the sum of variances among the presented strategies and demonstrated substantial efficiency gains over case‐control sampling.

Notably, across all sampling designs, GR led to a 92%–97% reduction in variance for ${\hat{\beta }}_{\mathrm{CD}4,\mathrm{Death}}$ but only a 6%–22% reduction for ${\hat{\beta }}_{\mathrm{CD}4,\mathrm{ADE}}$ (Table 5). This discrepancy is a direct consequence of the stark difference in measurement error levels in the Phase 1 variables, which were used to derive influence functions serving as raking variables in the GR analysis. These influence functions were strongly correlated with the true influence functions for the death model but not for the ADE model, making raking substantially more effective for the former. Accordingly, Table 5 shows that using an IPW‐optimal design between waves results in a notably higher sum of variances for raking estimators compared to the GR‐optimal approach. The GR‐based A‐optimal design, constructed by regressing Phase 2 influence function estimates on their Phase 1 counterparts, recognized that raking alone would yield large gains for ${\hat{\beta }}_{\mathrm{CD}4,\mathrm{death}}$ and thus prioritized minimizing variance for ${\hat{\beta }}_{\mathrm{CD}4,\mathrm{ADE}}$ through the sampling design by mimicking the univariate IPW‐optimal design for ADE (Supplemental Figure S1). This design most effectively addressed the imbalance in efficiency gains across parameters. This type of trade‐off does not arise in the single‐parameter setting, where Chen and Lumley [18] point out that IPW‐optimal designs are often approximately optimal for GR as well. Thus, this example demonstrates the heightened importance of using an A‐optimal design tailored to raking in the multiple‐parameter setting compared to the single‐parameter setting.

## 6 Discussion

This work presents a number of practical sampling strategies and examines key design considerations for two‐phase studies aimed at estimating multiple parameters with minimal variance. Through extensive numerical studies, we investigate the performance of these designs and demonstrate the strong benefits of adaptive, multiwave sampling using Neyman or Neyman–Wright allocation of influence functions and GR estimators. These simulations, supported by theoretical results in Section 3, also illustrate the utility of a novel application of A‐optimality to multiwave stratified sampling. A‐optimality shows a promising ability to minimize the sum of variances for both IPW and GR estimators across a wide range of measurement error and correlation scenarios. To support implementation of this approach, we have added an A‐optimal allocation feature to the R package ‘optimall’, available on CRAN [42]. While A‐optimality has previously been considered in a nonadaptive setting for IPW estimators, our derivation of an A‐optimal design specifically tailored to raking estimators and exploration of adaptive implementations are unique contributions of this work. Notably, we show that this approach can yield sampling strategies that offer substantial improvements over those optimized for IPW estimators, a result which differs from findings in the single‐parameter setting. The data example in Section 5 is just one illustration of this approach's practical strengths, particularly its ability to target sampling towards estimators for which the Phase 1 variables provide poor proxies for the variables of interest.

While A‐optimality effectively minimizes the sum of variances, our results also suggest that simplified approaches, such as independently optimizing allocation for each parameter in each wave or optimizing for one parameter per wave sequentially, often yield very similar estimator variances. A‐optimality's key advantage is its ability to assign explicit weights to different parameters, ensuring a more principled allocation. In contrast, independent allocation approaches (Strategies 3‐4) rely on the choice of arbitrary sample split points. This limitation is highlighted in the data example: when one parameter is harder to measure, simply splitting the Phase 2 sample in half does not lead to balanced efficiency gains. Similarly, in sequential strategies, later‐allocated parameters benefit disproportionately from earlier sampling waves. In both cases, an optimal split point likely exists, but it cannot be known during the design stage. A‐optimality naturally accounts for these complexities, making it the most practical and flexible strategy.

It should be noted that these simulations used only a simplified subset of the steps that could be taken to achieve an optimal sample design. Further improvements could be made by selecting stratum boundaries so that optimum allocation divides samples to strata more evenly, even subdividing strata between waves as necessary [8, 36]. Another avenue for design refinement is incorporating informative priors to improve the wave 1 allocation rather than relying solely on error‐prone Phase 1 data [37]. Multiple imputation could also be used to construct more efficient raking estimators, particularly in settings where the Phase 1 variables are poor surrogates for the Phase 2 variables of interest [11].

The potential for future work on efficient sampling designs for multiple outcomes is vast and increasingly important given the increasing use of EHRs in medical research [1]. One natural extension of this work would be to extend the simulations to a broader range of outcome models and incorporate design enhancements such as optimal stratification. Under these conditions, the advantages of alternative optimality criteria, such as those that account for parameter covariances, may become more apparent. Examples include D‐optimality, E‐optimality, I‐optimality, and the maximin relative‐efficiency criterion of Wang et al. [25] and [45, 46, 47]. Still, the practical advantages of A‐optimal designs with multiwave adaptive sampling and GR make it a compelling approach, and the findings of this work provide a foundation for future research on optimal design and estimation in studies using error‐prone or incomplete data.

## Funding

This work was supported by the U.S. National Institutes of Health (Grant Nos. R37‐AI131771 and P30‐AI110527) and the U.S. National Science Foundation Graduate Research Fellowship Program (Grant No. DGE‐2140004).

## Conflicts of Interest

The authors declare no conflicts of interest.

## Supporting information




**Figure S1:** Optimal stratum fractions for three strata defined on ADE or Death cases under A‐optimal allocation with respect to GR (GR), A‐optimal allocation with respect to IPW (IPW), univariate IPW optimal allocation with respect to $\beta_{\text{CD4, ADE}}$ (IPW‐ADE), and univariate IPW optimal allocation with respect to $\beta_{\text{CD4, Death}}$ (IPW‐Death). **Table S1:** Coefficients for data‐generating models in each simulation scenario. **Table S2:** Measurement error model parameters. Sens refers to sensitivity, the probability of the observed value being 1 if the true value is 1, and Spec to specificity, the probability of the true value being 0 if the true value is 0. The error model parameters for Z were held constant across measurement error cases for simplicity, with Sens(Z) = 0.9 and Spec(Z) = 0.95. **Table S3:** Empirical variance ($\times 10^{3}$) under Scenario 2O2P: Multiple predictors and Multiple outcomes of interest. **Table S4:** Empirical MSE ($\times 10^{3}$) under Scenario 2O: Multiple outcomes of interest. **Table S5:** Empirical MSE ($\times 10^{3}$) under Scenario 2P: Multiple predictors of interest. **Table S6:** Empirical MSE ($\times 10^{3}$) under Scenario 2O2P: Multiple predictors and Multiple outcomes of interest. **Table S7:** Empirical relative efficiency for sum of variances under IPW. **Table S8:** Coverage probabilities of estimated 95% confidence intervals for the simulation study for the two outcomes (2O) and two predictors (2P) scenarios. **Table S9:** Coverage probabilities of estimated 95% confidence intervals for the simulation study for the two outcomes and two predictors (2O2P) scenario. **Table S10:** Empirical variances ($\times 10^{3}$) for the A‐optimal strategy (Strategy 5) under varying priority weights in the two outcomes of interest and one predictor scenario with low measurement error and low correlation (2O‐A). **Table S11:** Empirical variance ($\times 10^{3}$) for each strategy under Scenario 2O: Two outcomes of interest in the supplemental scenario where the Phase 2 size is n=500. **Table S12:** Empirical variance ($\times 10^{3}$) for each strategy under Scenario 2P: Two predictors of interest in the supplemental scenario where the Phase 2 size is n=500. **Table S13:** Coverage probabilities of estimated 95% confidence intervals for the simulation study for the two outcomes (2O) and two predictors (2P) scenarios in the supplemental scenario where the Phase 2 size is n=500.

## Data Availability

Data from the example in this paper may be obtained by contacting the corresponding author and appropriate data use agreements. Data are not publicly available due to privacy restrictions.
