# Supplementary material for: Optimal Two‐Phase Sampling Designs for Generalized Raking Estimators With Multiple Parameters of Interest
Source: Stat Med. 2026 Oct 16;45(23-24):e70786. doi: 10.1002/sim.70786 (PMC13675120; doi:10.1002/sim.70786)
Supplement: Supplementary file 1 [file SIM-45-0-s001.pdf]

Supplementary Materials for "Optimal two-phase sampling designs for generalized raking estimators with multiple parameters of interest"

Jasper B. Yang[1], Bryan E. Shepherd[2], Thomas Lumley[3], and Pamela A. Shaw[1,4]

[1]Department of Biostatistics, University of Washington, Seattle, WA, USA
[2]Department of Biostatistics, Vanderbilt University, Nashville, Tennessee, USA
[3]Department of Statistics, University of Auckland, Auckland, New Zealand
[4]Biostatistics Division, Kaiser Permanente Washington Health Research Institute, Seattle, WA, USA

September 15, 2026

Contents

S1 Proofs **2**
S1.1 Proof of exact integer-valued A-optimal allocation algorithm . . . 2
S1.2 Proof of convergence of Algorithm 1 to oracle optimal allocation . . . 2

S2 Simulation Set-Up **4**
S2.1 Data generation . . . 4
S2.2 Measurement error . . . 4
S2.3 Further details on design strategies 1-5 . . . 4
S2.4 Generalized raking procedure . . . 5
S2.5 Variance estimation . . . 5

S3 Supplemental Figures **6**

S4 Supplemental Tables **7**

Supplemental Materials

S1 Proofs

S1.1 Proof of exact integer-valued A-optimal allocation algorithm

We seek an integer-valued allocation that satisfies

$$\underset{n_1,\ldots,n_K}{\operatorname{argmin}} \sum_{p=1}^{P} \left[a_p \sum_{k=1}^{K} \frac{N_k^2}{n_k} \sigma_{p,k}^2 \right] = \underset{n_1,\ldots,n_K}{\operatorname{argmin}} \sum_{k=1}^{K} \frac{N_k^2 \sum_{p=1}^{P} a_p \sigma_{p,k}^2}{n_k},$$

as well as the constraints $n_k \leq N_k$ and $n_k \geq 2$ for all $k = 1, ..., K$. Note that $1 - \frac{1}{n_k}$ can be re-written as a telescoping series $(1 - \frac{1}{2}) + (\frac{1}{2} - \frac{1}{3}) + \cdots + (\frac{1}{n_k - 1} - \frac{1}{n_k}) = \frac{1}{1 \cdot 2} + \frac{1}{2 \cdot 3} + \cdots + \frac{1}{(n_k - 1) \cdot n_k}$. Then,

$$\begin{aligned}
\sum_{k=1}^{K} \frac{N_k^2 \sum_{p=1}^{P} a_p \sigma_{p,k}^2}{n_k} &= \sum_{k=1}^{K} \left[\left(N_k^2 \sum_{p=1}^{P} a_p \sigma_{p,k}^2 \right) \left(1 - \frac{1}{1 \cdot 2} - \frac{1}{2 \cdot 3} - \cdots - \frac{1}{(n_k - 1) \cdot n_k} \right) \right] \\
&= \sum_{k=1}^{K} \left(N_k^2 \sum_{p=1}^{P} a_p \sigma_{p,k}^2 \right) \\
&\quad - \sum_{k=1}^{K} \left(\frac{N_k^2 \sum_{p=1}^{P} a_p \sigma_{p,k}^2}{1 \cdot 2} + \cdots + \frac{N_k^2 \sum_{p=1}^{P} a_p \sigma_{p,k}^2}{(n_k - 1) \cdot n_k} \right).
\end{aligned} \tag{S1}$$

This expression, a weighted sum of variances, is minimized when the second sum in the difference is maximized. Thus, the optimum allocation occurs when the n_k are chosen so that the n largest terms are included in the second sum. For unbiased estimates of variance, we require that $n_k \geq 2$ for all k, so optimum allocation with this added constraint involves choosing the $n - 2K$ largest values from the array of priority values (note that the terms from Equation S1 are square-rooted in (S2), which preserves the relative priorities but makes clear the similarity to Wright's original version):

$$\begin{array}{cccc}
\frac{N_1 \sqrt{\sum_{p=1}^{P} a_p \sigma_{p,1}^2}}{\sqrt{2 \cdot 3}} & \frac{N_1 \sqrt{\sum_{p=1}^{P} a_p \sigma_{p,1}^2}}{\sqrt{3 \cdot 4}} & \frac{N_1 \sqrt{\sum_{p=1}^{P} a_p \sigma_{p,1}^2}}{\sqrt{4 \cdot 5}} & \cdots \\
& \vdots & & \\
\frac{N_k \sqrt{\sum_{p=1}^{P} a_p \sigma_{p,k}^2}}{\sqrt{2 \cdot 3}} & \frac{N_k \sqrt{\sum_{p=1}^{P} a_p \sigma_{p,k}^2}}{\sqrt{3 \cdot 4}} & \frac{N_k \sqrt{\sum_{p=1}^{P} a_p \sigma_{p,k}^2}}{\sqrt{4 \cdot 5}} & \cdots \\
& \vdots & & \\
\frac{N_K \sqrt{\sum_{p=1}^{P} a_p \sigma_{p,K}^2}}{\sqrt{2 \cdot 3}} & \frac{N_K \sqrt{\sum_{p=1}^{P} a_p \sigma_{p,K}^2}}{\sqrt{3 \cdot 4}} & \frac{N_K \sqrt{\sum_{p=1}^{P} a_p \sigma_{p,K}^2}}{\sqrt{4 \cdot 5}} & \cdots.
\end{array} \tag{S2}$$

The number of samples assigned to stratum k is equal to 2 plus the number of priority values it has among the largest $n - 2K$ in (S2).

S1.2 Proof of convergence of Algorithm 1 to oracle optimal allocation

Consider an asymptotic regime where $T \geq 2$ is a fixed number of waves, K is a fixed number of strata, and $n \to \infty$ with $n/N \to \gamma \in (0, 1)$. Further, let the wave 1 size $n^{(1)} \to \infty$ but $n^{(1)}/n \to 0$, and assume that the wave 1 design is such that $\forall k$, the wave 1 stratum sample sizes $n_k^{(1)} \to \infty$, which occurs for instance when Step 5 of Algorithm 1 is used and the true $\sigma_{p,k}^{h^*}$ is non-zero for at least one p for every k.

Consider the term which determines the wave 2 allocation,

$$\hat{s}_k^{(1)} = \sqrt{\sum_{p=1}^{P} a_p \left\{ \hat{\sigma}_{p,k}^{(\epsilon,1)} \right\}^2},$$

and the corresponding quantity defining the oracle optimal allocation,

$$s_k = \sqrt{\sum_{p=1}^{P} a_p \left\{\sigma_{p,k}^{(\epsilon)}\right\}^2},$$

where $\left\{\sigma_{p,k}^{(\epsilon)}\right\}^2 = \text{Var}[\epsilon_{i,p}|\text{stratum k}]$ is the true within-stratum residualized variance. As demonstrated in Section 3.3 of the main text, the optimal Neyman allocation, which is asymptotically equivalent to the optimal integer-valued allocation up to rounding, selects $n_k/n = N_k s_k / \sum_{k'} N_{k'} s_{k'}$.

In algorithm 1, $\left\{\hat{\sigma}_{p,k}^{(\epsilon,1)}\right\}^2$ is the estimated residual variance from a least-squares regression of the estimated influence function $\hat{h}_{i,p}$ on the (possibly estimated) raking variable $\hat{\mathbf{c}}_{i,p}$. To clearly state the convergence requirements, we can more specifically write $\hat{h}_{i,p} := \hat{h}_p(Z_i)$ and similarly $\hat{\mathbf{c}}_{i,p} := \hat{\mathbf{c}}_p(Z_i)$, where Z_i is the observed data for unit i. Then $\hat{h}_p$ is an estimator for the true influence function h_p, defined under the true data-generating law $\mathbb{P}_0$, and $\hat{\mathbf{c}}_p$ is an estimator for the true raking variable $\mathbf{c}_p$, which may itself involve unknown features of $\mathbb{P}_0$, as is the case for the optimal raking variable. Next we introduce conditions on these quantities so that the OLS regression of $\hat{h}_p$ on $\hat{\mathbf{c}}_p$ produces residuals whose variance converges to the population residual variance. We assume first that for each p, k, $\|\hat{h}_p - h_p\|_{L_2(\mathbb{P}_{0,k})} = o_p(1)$ and $\|\hat{\mathbf{c}}_p - \mathbf{c}_p\|_{L_2(\mathbb{P}_{0,k})} = o_p(1)$, where $\mathbb{P}_{0,k}$ is the distribution in stratum k and $\|\cdot\|_{L_2(\cdot)}$ is the L_2 norm under $\mathbb{P}_0$. These conditions state that the estimated influence function (which may depend on nuisance parameters) and estimated raking variable appropriately converge to their population-level truths. When $\mathbf{c}_{i,p}$ is directly observed in Phase 1, then the second condition is automatic.

When $\hat{\mathbf{c}}_p$ is estimated using phase 2 data, we additionally require a sample-splitting condition, assuming $\hat{\mathbf{c}}_p$ and $\hat{h}_p$ are estimated using different data folds. Let $\mathcal{A}$ and $\mathcal{B}$ denote these folds, where fold $\mathcal{A}$ is used to generate $\hat{\mathbf{c}}_p$ and fold $\mathcal{B}$ is used to generate $\hat{h}_p$. We assume that number of samples in each stratum in each fold tends to ∞, except in the case where $\hat{\mathbf{c}}_p$ is not estimated using phase 2 data, where $\mathcal{B}$ becomes the entire phase 2 sample and $\mathcal{A} = \emptyset$. Then, we have for each p,

$$(\hat{\alpha}_p, \hat{\theta}_p) = \arg\min_{\alpha,\theta} \frac{1}{|\mathcal{B}|} \sum_{i\in\mathcal{B}} w_i \left\{\hat{h}_p(Z_i) - \alpha - \theta^\top \hat{\mathbf{c}}_p(Z_i)\right\}^2,$$

where w_i are IPW weights reflecting both phase 2 sampling and assignment to fold $\mathcal{B}$. Under standard weighted least-squares regularity conditions, we have $(\hat{\alpha}_p, \hat{\theta}_p) \xrightarrow{p} (\alpha_p, \theta_p)$.

Next, define the estimated and population-level OLS residuals

$$\hat{\epsilon}_{i,p} = \hat{h}_p(Z_i) - \hat{\alpha}_p - \hat{\theta}_p^\top \hat{\mathbf{c}}_p(Z_i),$$

and

$$\epsilon_{i,p} = h_p(Z_i) - \alpha_p - \theta_p^\top \mathbf{c}_p(Z_i),$$

respectively. Together with the assumed L_2 convergence of $\hat{h}_p$ and $\hat{\mathbf{c}}_p$, consistency of $\hat{\alpha}$ and $\hat{\theta}$ implies that

$$\|\hat{\epsilon}_p - \epsilon_p\|_{L_2(\mathbb{P}_{0,k})} = o_p(1),$$

because

$$\|\hat{\epsilon}_p - \epsilon_p\|_{L_2(\mathbb{P}_{0,k})} \le \|\hat{h}_p - h_p\|_{L_2(\mathbb{P}_{0,k})} + |\hat{\alpha}_p - \alpha_p| + \|\hat{\theta}_p - \theta_p\|\|\mathbf{c}_p\|_{L_2(\mathbb{P}_{0,k})} + \|\hat{\theta}_p\|\|\hat{\mathbf{c}}_p - \mathbf{c}_p\|_{L_2(\mathbb{P}_{0,k})}$$

Then, noting that the weights w_i are constant within each stratum, we have by the weak law of large numbers and continuous mapping theorem that $\frac{1}{|\mathcal{B}_k|}\sum_{i\in\mathcal{B}_k} \hat{\epsilon}_{i,p} \xrightarrow{p} \mathbb{E}[\epsilon_{i,p}|\text{stratum k}]$ and $\frac{1}{|\mathcal{B}_k|}\sum_{i\in\mathcal{B}_k} \hat{\epsilon}_{i,p}^2 \xrightarrow{p} \mathbb{E}[\epsilon_{i,p}^2|\text{stratum k}]$, where $\mathcal{B}_k$ is the set of units in stratum k in fold $\mathcal{B}$. Hence, the proposed estimator

$$\left\{\hat{\sigma}_{p,k}^{(\epsilon,1)}\right\}^2 = \frac{1}{|\mathcal{B}_k|}\sum_{i\in\mathcal{B}_k} \hat{\epsilon}_{i,p}^2 - \left(\frac{1}{|\mathcal{B}_k|}\sum_{i\in\mathcal{B}_k} \hat{\epsilon}_{i,p}\right)^2 \xrightarrow{p} \{\sigma_{p,k}^{(\epsilon)}\}^2.$$

Hence, by the continuous mapping theorem, $\hat{s}_k^{(1)} \xrightarrow{p} s_k$, and since the Neyman allocation is a continuous function of $\hat{s}_k^{(1)}$, the wave 2 A-optimal (Neyman) allocation for stratum k converges in probability to the oracle A-optimal (Neyman) allocation. These optimal Neyman allocations are asymptotically equivalent to the optimal integer-valued allocation up to rounding. This convergence also follows for the following waves $t = 3, ..., T$, meaning that under the given asymptotic regime, only wave 1 is collected with a sub-optimal allocation. However, $n^{(1)}/n \to 0$, so its contribution to the overall phase 2 sampling design is asymptotically negligible.

S2 Simulation Set-Up

S2.1 Data generation

For every individual $i = 1, 2, \ldots, N$, covariates X_1, X_2, and Z were simulated from a multivariate normal distribution with mean vector (0,0,0) and covariance matrix

$$\begin{pmatrix} 1 & \text{cor}(X_1, X_2) & 0.10 \\ \text{cor}(X_1, X_2) & 1 & 0.25 \\ 0.10 & 0.25 & 1 \end{pmatrix}.$$

Then, Z was dichotomized into a binary variable and later standardized to have mean zero and variance 1. The two binary outcomes of interest, Y_1 and Y_2 were then generated according to the logistic models in Equation (10). The coefficients varied by scenario and are presented in Supplemental Table S1.

S2.2 Measurement error

For each dataset, we assumed that error-prone or proxy versions of the variables X_1, X_2, Z, Y_1, and Y_2, denoted X_1^*, X_2^*, Z^*, Y_1^*, and Y_2^* respectively, were available in the phase 1 data. For the continuous variables X_1 and X_2, phase 1 versions were generated following the classical measurement error models $X_1^* = X_1 + U_{X_1}$ and $X_2^* = X_2 + U_{X_2}$ respectively with U_{X_1} and U_{X_2} drawn from a multivariate normal distribution with mean zero. For binary variables Z, Y_1, and Y_2, phase 1 versions were generated with pre-specified sensitivity and specificity. We simulated all of these errors with correlation to reflect the reality of error-prone data. We considered low and high degrees of measurement error for the outcome and exposure(s) of interest. The error-generating model parameters for Y_1^*, Y_2^*, X_1^* and X_2^* varied in each case, as shown in Supplemental Table S2.

S2.3 Further details on design strategies 1-5

The exact form of Strategies 1-5 varied depending on the simulation scenario:

- Scenario 2O: Two outcomes, one covariate of interest
  - **Strategy 1:** Case-control sampling in a single wave. $n/4$ cases of Y_1^* and Y_2^*, $n/4$ controls of Y_1^* and cases of Y_2^*, $n/4$ cases of Y_1^* and controls of Y_2^*, and $n/4$ controls of Y_1^* and Y_2^* were selected for sampling in a single wave.
  - **Strategy 2:** Independent allocation for each parameter in four equally sized waves under the simultaneous approach. $n_{(t)}$ samples were collected at each wave, with $n_{(t)}/2$ allocated optimally with respect to β_{11} and $n_{(t)}/2$ with respect to β_{12}.
  - **Strategy 3:** Independent allocation for each parameter over four equally sized waves under the sequential approach. Waves 1 and 2 were allocated optimally with respect to β_{11} and waves 3 and 4 with respect to β_{12}.
  - **Strategy 4:** Same as Strategy 3, only Waves 1 and 2 were allocated optimally with respect to β_{12} and waves 3 and 4 with respect to β_{11}.
  - **Strategy 5:** Weighted A-optimality over four equally sized waves.
- Scenario 2P: One outcome, two covariates of interest
  - **Strategy 1:** Case-control sampling in a single wave. $n/2$ cases of Y_2^* and $n/2$ controls of Y_2^* were selected for sampling in a single wave.
  - **Strategy 2:** Independent allocation for each parameter in four equally sized waves under the simultaneous approach. $n_{(t)}$ samples were collected at each wave, with $n_{(t)}/2$ allocated optimally with respect to β_{12} and $n_{(t)}/2$ with respect to β_{22}.
  - **Strategy 3:** Independent allocation for each parameter over four equally sized waves under the sequential approach. Waves 1 and 2 were allocated optimally with respect to β_{12} and waves 3 and 4 with respect to β_{22}.
  - **Strategy 4:** Same as Strategy 3, only Waves 1 and 2 were allocated optimally with respect to β_{22} and waves 3 and 4 with respect to β_{12}.

  - **Strategy 5:** Weighted A-optimality over four equally sized waves.

- Scenario 2O2P: Two outcomes, two covariates of interest
  - **Strategy 1:** Case-control sampling in a single wave. $n/4$ cases of Y_1^* and Y_2^*, $n/4$ controls of Y_1^* and cases of Y_2^*, $n/4$ cases of Y_1^* and controls of Y_2^*, and $n/4$ controls of Y_1^* and Y_2^* were selected for sampling in a single wave.
  - **Strategy 2:** Independent allocation for each parameter in four equally sized waves under the simultaneous approach. $n_{(t)}$ samples were collected at each wave, with $n_{(t)}/4$ allocated optimally with respect to β_{11}, $n_{(t)}/4$ with respect to β_{12}, $n_{(t)}/4$ with respect to β_{21}, and $n_{(t)}/4$ with respect to β_{22}.
  - **Strategy 3:** Independent allocation for each parameter over four equally sized waves under the sequential approach. Wave 1 was allocated optimally with respect to β_{11}, wave 2 was allocated optimally with respect to β_{21}, wave 3 was allocated optimally with respect to β_{12}, and wave 4 with respect to β_{22}.
  - **Strategy 4:** No strategy 4 in this case. Only one order was considered instead of all possible orderings of the four coefficients
  - **Strategy 5:** Weighted A-optimality over four equally sized waves.

For strategies 2-5, strata were constructed using the error prone phase 1 variables Y_1^*, Y_2^*, X_1^*, and X_2^*. For each scenario, the error-prone versions of the binary outcome(s) were first used to define groups. Specifically, in Scenario 2P, the two initial groups were defined by $Y_2^* = 1$ and $Y_2^* = 0$, while in Scenarios 2O and 2O2P, there were four initial groups defined by combinations of the binary Y_1^* and Y_2^*. Final strata were then defined by dividing the initial groups based on quantiles of the error-prone covariate(s). In Scenario 2O, each group was split at the local (i.e. within-group) 0.25 and 0.75 quantiles of X_1^*, resulting in $4 \times 3 = 12$ strata. For Scenarios 2P and 2O2P, each group was split at the local medians of X_1^* and X_2^* successively, resulting in $2^3 = 8$ and $4 \times 2 \times 2 = 16$ strata respectively. These quantile-based constructions were chosen as practical approximations to optimal stratification motivated by Särndal et al. (2003) and Amorim et al. (2021), who show that optimal stratum boundaries lead to Neyman allocation assigning approximately equal samples to each stratum, and also taking into consideration the potential for the stability issues in variance estimation noted in Section 3.5 if the number of strata were too large. More sophisticated approaches to optimal stratification are beyond the scope of this work.

S2.4 Generalized raking procedure

For the generalized raking procedures in the simulation study and data example, the vector of raking variables $\mathbf{c}_{i,}$ includes the influence functions for each coefficient in the target model fit using the error-prone phase 1 data. While theoretically, this may not be as efficient as using multiple imputation to approximate the optimal raking variable, it is more straightforward to implement, and several studies have found that it often led to similar performance in finite samples (Oh et al., 2021; Han et al., 2021; Shepherd et al., 2023). In computing the optimal raking design at each wave, we follow the procedures of Section 3.4 with these raking variables as $\mathbf{c}_{i,p}$ and estimates for the true influence functions $\mathbf{h_i}$ computed as (unweighted) influence functions from the GR estimator using all phase 2 data that has been collected up to that point.

S2.5 Variance estimation

Variance estimates, which were used to generate estimated confidence intervals whose coverages were reported in Supplemental Table S8, were computed using the standard two-phase variance estimation procedures in the R package 'survey'. We briefly outline these procedures here, noting that the replication code contains the exact steps to compute these estimates, and referring to Binder (1983), Lumley (2004), Breslow et al. (2009), and Lumley (2011) for further technical details.

The core idea of variance estimation for asymptotically linear estimators in two-phase samples comes from the law of total variance, conditioning on the realized phase 1 sample. For $\hat{\boldsymbol{\beta}} \in \{\hat{\boldsymbol{\beta}}_{\text{IPW}}, \hat{\boldsymbol{\beta}}_{\text{GR}}\}$, we have

$$\text{Var}\left(\hat{\boldsymbol{\beta}}\right) = \text{Var}\left(\mathbb{E}\left[\hat{\boldsymbol{\beta}}\middle|\text{phase 1 sample}\right]\right) + \mathbb{E}\left[\text{Var}\left(\hat{\boldsymbol{\beta}}\middle|\text{phase 1 sample}\right)\right]$$

For the first term in the sum, $\text{Var}\left(\mathbb{E}\left[\hat{\boldsymbol{\beta}}\middle|\text{phase 1 sample}\right]\right)$, we note that both $\hat{\boldsymbol{\beta}}_{\text{IPW}}$ and $\hat{\boldsymbol{\beta}}_{\text{GR}}$ are asymptotically unbiased estimators for $\tilde{\beta}$, the ideal estimator that would have been used if all data were available for the phase 1 cohort, conditional on the phase 1 sample. Hence we can approximate this first term with $\text{Var}(\tilde{\beta})$ and estimate it

using an inverse-probability weighted version of the standard influence-function-based variance approximation. The second term, $\mathbb{E}\left[\text{Var}\left(\hat{\boldsymbol{\beta}}|\text{phase 1 sample}\right)\right]$, represents the variance due to the second phase of sampling. It can be estimated using standard tools for estimating the variance of an estimator for a finite-population mean due to the asymptotic representation in Equation (3) of the main text. The `twophase()` function in the survey package can be used to implement these procedures.

In the adaptive multiwave sampling setting, an additional consideration for variance estimation is that the sampling probabilities in later waves are dependent on samples collected in previous waves. In this work, we address this complication using post-stratification, as described in Yang et al. (2025) and used in the motivating example of Shepherd et al. (2023). This approach uses the final observed n_k/N_k in each stratum as the sampling probability assigned to each unit at the analysis stage, and the results of Supplemental Table S13 demonstrate that it led to good coverage. Recently, Kluger and Bates (2026) proposed an alternative approach that explicitly uses conditional probabilities in a non-stratified but adaptive setting. Extending it to the present setting is left for future work.

S3 Supplemental Figures

Supplemental Figure S1: Optimal stratum fractions for three strata defined on ADE or Death cases under A-optimal allocation with respect to GR (GR), A-optimal allocation with respect to IPW (IPW), univariate IPW optimal allocation with respect to $\beta_{\text{CD4, ADE}}$ (IPW-ADE), and univariate IPW optimal allocation with respect to $\beta_{\text{CD4, Death}}$ (IPW-Death).

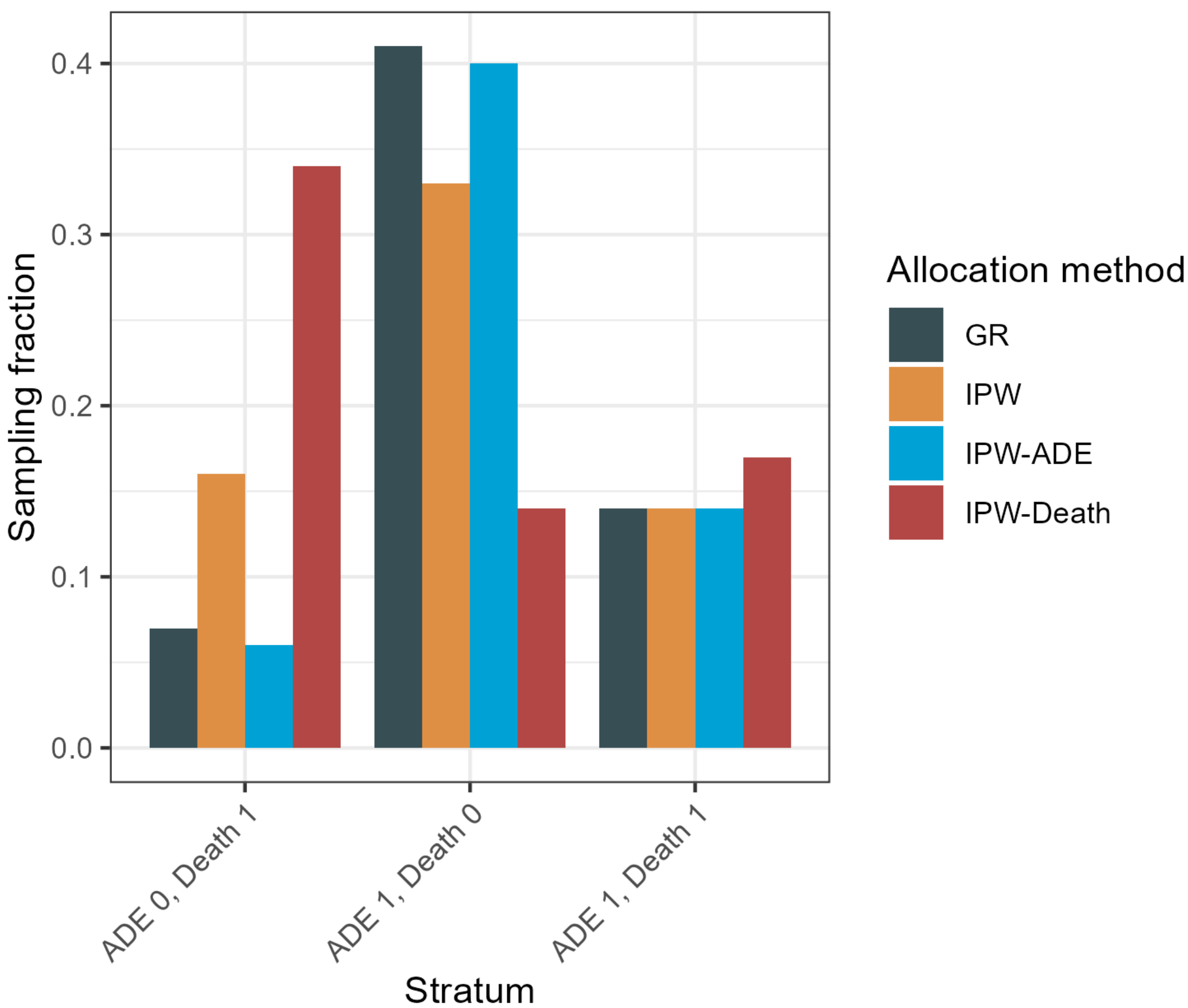

S4 Supplemental Tables

Supplemental Table S1: Coefficients for data-generating models in each simulation scenario.

	Outcome 1					Outcome 2					
Scenarios	β_{01}	β_{11}	β_{21}	β_{31}	β_{41}	β_{02}	β_{12}	β_{22}	β_{32}	$\text{cor}(Y_1, Y_2)$	$\text{cor}(X_1, X_2)$
2O-A, 2O-B	-1.5	0.4	0	0.3	0	-0.5	0.2	0.5	0	0.02	-
2O-C, 2O-D	-3.1	0.4	1.0	0.7	1.9	-0.8	0.2	1.3	0.8	0.44	-
2P-A, 2P-B	-	-	-	-	-	-2.1	0.3	0.7	0.7	-	0.05
2P-C, 2P-D	-	-	-	-	-	-2.1	0.3	0.7	0.7	-	0.65
202P-A, 2O2P-B	-1.5	0.4	0.6	0.3	0.3	-2.1	0.3	0.7	0.7	0.16	0.3

Supplemental Table S2: Measurement error model parameters. Sens refers to sensitivity, the probability of the observed value being 1 if the true value is 1, and Spec to specificity, the probability of the true value being 0 if the true value is 0. The error model parameters for Z were held constant across measurement error cases for simplicity, with $\text{Sens}(Z) = 0.9$ and $\text{Spec}(Z) = 0.95$.

Scenarios	$\text{Sens}(Y_1)$	$\text{Spec}(Y_1)$	$\text{Sens}(Y_2)$	$\text{Spec}(Y_2)$	$\text{Var}(U_{X_1})$	$\text{Var}(U_{X_2})$
2O-A, 2O-C	0.95	0.99	0.9	0.95	0.15	0.1
2O-B, 2O-D	0.85	0.90	0.8	0.85	0.5	0.1
2P-A, 2P-C	-	-	0.90	0.95	0.15	0.5
2P-B, 2P-D	-	-	0.85	0.90	0.4	0.6
2O2P-A	0.95	0.99	0.90	0.95	0.15	0.5
2O2P-B	0.85	0.90	0.80	0.85	0.4	0.6

Supplemental Table S3: Empirical variance ($\times 10^3$) under Scenario 2O2P: Multiple predictors and Multiple outcomes of interest.

**Error level**	**Estim-ator**		**Strat 1: Case-control**	**Strat 2: Simult-aneous**	**Strat 3: Sequential**	**Strat 5: A-optimal**
Low (A)	IPW	$\text{Var}(\hat{\beta}_{11})$	7.82	4.19	4.66	3.95
		$\text{Var}(\hat{\beta}_{21})$	8.84	5.48	6.19	5.40
		$\text{Var}(\hat{\beta}_{12})$	7.15	5.25	5.59	5.08
		$\text{Var}(\hat{\beta}_{22})$	8.85	6.53	7.24	6.54
	GR	$\text{Var}(\hat{\beta}_{11})$	2.91	2.50	2.81	2.46
		$\text{Var}(\hat{\beta}_{21})$	5.23	4.08	4.52	4.19
		$\text{Var}(\hat{\beta}_{12})$	4.68	3.87	3.85	3.92
		$\text{Var}(\hat{\beta}_{22})$	6.36	5.88	5.32	5.53
High (B)	IPW	$\text{Var}(\hat{\beta}_{11})$	8.03	5.76	6.60	6.16
		$\text{Var}(\hat{\beta}_{21})$	9.19	6.82	7.53	6.91
		$\text{Var}(\hat{\beta}_{12})$	9.30	7.60	7.76	7.29
		$\text{Var}(\hat{\beta}_{22})$	10.46	8.84	9.53	8.70
	GR	$\text{Var}(\hat{\beta}_{11})$	5.73	5.07	5.77	5.20
		$\text{Var}(\hat{\beta}_{21})$	7.30	6.13	6.85	6.22
		$\text{Var}(\hat{\beta}_{12})$	6.87	7.28	6.66	6.78
		$\text{Var}(\hat{\beta}_{22})$	8.61	8.16	8.40	8.83

Supplemental Table S4: Empirical MSE ($\times 10^3$) under Scenario 2O: Multiple outcomes of interest.

**Corr level**	**Error level**	**Estim-ator**		**Strat 1: Case-control**	**Strat 2: Simult-aneous**	**Strat 3: β_{12} last**	**Strat 4: β_{11} last**	**Strat 5: A-optimal**
Low	Low (A)	IPW	MSE($\hat{\beta}_{11}$)	5.59	2.57	3.06	2.63	2.73
			MSE($\hat{\beta}_{12}$)	6.18	2.74	2.42	3.19	2.53
		GR	MSE($\hat{\beta}_{11}$)	2.24	1.94	2.13	1.91	1.94
			MSE($\hat{\beta}_{12}$)	2.61	2.11	1.95	2.50	2.15
	High (B)	IPW	MSE($\hat{\beta}_{11}$)	5.76	5.03	5.57	4.73	4.88
			MSE($\hat{\beta}_{12}$)	5.30	3.88	3.72	4.25	3.63
		GR	MSE($\hat{\beta}_{11}$)	4.56	4.50	5.13	4.51	4.63
			MSE($\hat{\beta}_{12}$)	4.06	3.58	3.39	3.98	3.63
High	Low (C)	IPW	MSE($\hat{\beta}_{11}$)	7.44	4.30	4.47	4.09	4.11
			MSE($\hat{\beta}_{12}$)	7.48	3.36	3.30	4.28	3.42
		GR	MSE($\hat{\beta}_{11}$)	3.55	2.97	3.11	2.91	2.88
			MSE($\hat{\beta}_{12}$)	3.36	2.67	2.40	3.00	2.68
	High (D)	IPW	MSE($\hat{\beta}_{11}$)	8.64	7.70	7.98	7.69	7.60
			MSE($\hat{\beta}_{12}$)	6.76	4.99	4.56	5.47	4.90
		GR	MSE($\hat{\beta}_{11}$)	7.12	7.12	7.59	6.80	6.60
			MSE($\hat{\beta}_{12}$)	5.04	4.63	4.23	5.23	4.45

Supplemental Table S5: Empirical MSE ($\times 10^3$) under Scenario 2P: Multiple predictors of interest.

**Corr level**	**Error level**	**Estim-ator**		**Strat 1: Case-control**	**Strat 2: Simult-aneous**	**Strat 3: β_{12} last**	**Strat 4: β_{11} last**	**Strat 5: A-optimal**
Low	Low (A)	IPW	MSE($\hat{\beta}_{12}$)	6.38	4.74	4.86	4.79	4.71
			MSE($\hat{\beta}_{22}$)	8.20	6.32	6.01	6.09	6.04
		GR	MSE($\hat{\beta}_{12}$)	3.92	3.68	3.54	3.85	3.62
			MSE($\hat{\beta}_{22}$)	5.83	5.43	5.47	5.40	5.28
	High (B)	IPW	MSE($\hat{\beta}_{12}$)	6.95	6.01	5.71	6.20	6.00
			MSE($\hat{\beta}_{22}$)	8.45	7.38	7.68	7.09	7.76
		GR	MSE($\hat{\beta}_{12}$)	5.59	5.26	5.18	5.52	5.28
			MSE($\hat{\beta}_{22}$)	7.00	7.35	7.70	6.78	6.74
High	Low (C)	IPW	MSE($\hat{\beta}_{12}$)	11.14	8.56	8.90	9.10	9.01
			MSE($\hat{\beta}_{22}$)	12.71	9.92	10.65	10.05	10.20
		GR	MSE($\hat{\beta}_{12}$)	7.73	7.18	6.95	6.93	6.87
			MSE($\hat{\beta}_{22}$)	10.10	9.27	9.07	8.63	8.74
	High (D)	IPW	MSE($\hat{\beta}_{12}$)	11.51	10.46	10.88	11.00	10.67
			MSE($\hat{\beta}_{22}$)	13.57	12.12	13.03	11.97	11.84
		GR	MSE($\hat{\beta}_{12}$)	9.35	9.65	9.49	9.71	9.71
			MSE($\hat{\beta}_{22}$)	11.54	11.39	11.84	11.17	11.19

Supplemental Table S6: Empirical MSE ($\times 10^3$) under Scenario 2O2P: Multiple predictors and Multiple outcomes of interest.

**Error level**	**Estim-ator**		**Strat 1: Case-control**	**Strat 2: Simult-aneous**	**Strat 3: Sequential**	**Strat 5: A-optimal**
Low (A)	IPW	MSE($\hat{\beta}_{11}$)	7.82	4.23	4.67	3.98
		MSE($\hat{\beta}_{21}$)	8.89	5.69	6.38	5.61
		MSE($\hat{\beta}_{12}$)	7.15	5.31	5.63	5.11
		MSE($\hat{\beta}_{22}$)	8.89	6.87	8.21	7.05
	GR	MSE($\hat{\beta}_{11}$)	2.92	2.50	2.81	2.47
		MSE($\hat{\beta}_{21}$)	5.25	4.18	4.61	4.27
		MSE($\hat{\beta}_{12}$)	4.68	3.89	3.86	3.96
		MSE($\hat{\beta}_{22}$)	6.44	6.30	5.97	5.94
High (B)	IPW	MSE($\hat{\beta}_{11}$)	8.06	5.92	6.65	6.28
		MSE($\hat{\beta}_{21}$)	9.21	7.05	7.80	7.14
		MSE($\hat{\beta}_{12}$)	9.30	7.78	8.12	7.44
		MSE($\hat{\beta}_{22}$)	10.52	9.52	11.39	9.43
	GR	MSE($\hat{\beta}_{11}$)	5.75	5.11	5.81	5.30
		MSE($\hat{\beta}_{21}$)	7.31	6.35	7.06	6.42
		MSE($\hat{\beta}_{12}$)	6.88	7.45	6.77	6.93
		MSE($\hat{\beta}_{22}$)	8.65	8.88	9.54	9.54

Supplemental Table S7: Empirical relative efficiency for sum of variances under IPW.

**Scenario**		**Corr level**	**Error level**	**Strat 1: Case-control**	**Strat 2: Simult-aneous**	**Strat 3: Sequential order 1**	**Strat 4: Sequential order 2**	**Strat 5: A-optimal**
2O	A	Low	Low	0.45	1.00	0.96	0.91	1.00
	B		High	0.76	0.96	0.92	0.96	1.00
	C	High	Low	0.50	0.99	0.97	0.90	1.00
	D		High	0.80	0.98	0.99	0.96	1.00
2P	A	Low	Low	0.72	0.98	1.00	0.98	1.00
	B		High	0.86	1.03	1.06	1.02	1.00
	C	High	Low	0.80	1.04	0.99	1.00	1.00
	D		High	0.88	1.00	0.95	0.97	1.00
2O2P	A	Moderate	Low	0.64	0.98	0.89	-	1.00
	B		High	0.79	1.00	0.92	-	1.00

Supplemental Table S8: Coverage probabilities of estimated 95% confidence intervals for the simulation study for the two outcomes (2O) and two predictors (2P) scenarios.

**Scen-ario**	**Corr level**	**Error level**	**Estim-ator**		**Strat 1: Case-control**	**Strat 2: Simult-aneous**	**Strat 3: Sequential order 1**	**Strat 4: Sequential order 2**	**Strat 5: A-optimal**
2O	Low	Low (A)	IPW	$\hat{\beta}_{11}$	0.94	0.95	0.95	0.94	0.95
				$\hat{\beta}_{12}$	0.94	0.94	0.94	0.94	0.94
			GR	$\hat{\beta}_{11}$	0.95	0.95	0.95	0.94	0.94
				$\hat{\beta}_{12}$	0.95	0.93	0.94	0.93	0.92
		High (B)	IPW	$\hat{\beta}_{11}$	0.96	0.94	0.94	0.94	0.94
				$\hat{\beta}_{12}$	0.95	0.95	0.94	0.95	0.95
			GR	$\hat{\beta}_{11}$	0.96	0.95	0.95	0.93	0.95
				$\hat{\beta}_{12}$	0.95	0.94	0.94	0.94	0.94
	High	Low (C)	IPW	$\hat{\beta}_{11}$	0.94	0.94	0.95	0.94	0.95
				$\hat{\beta}_{12}$	0.96	0.94	0.94	0.94	0.95
			GR	$\hat{\beta}_{11}$	0.95	0.95	0.95	0.94	0.95
				$\hat{\beta}_{12}$	0.95	0.94	0.94	0.94	0.93
		High (D)	IPW	$\hat{\beta}_{11}$	0.95	0.94	0.95	0.93	0.94
				$\hat{\beta}_{12}$	0.96	0.93	0.94	0.94	0.93
			GR	$\hat{\beta}_{11}$	0.95	0.94	0.94	0.93	0.94
				$\hat{\beta}_{12}$	0.95	0.94	0.94	0.93	0.94
2P	Low	Low (A)	IPW	$\hat{\beta}_{11}$	0.95	0.94	0.94	0.94	0.94
				$\hat{\beta}_{12}$	0.94	0.94	0.94	0.95	0.94
			GR	$\hat{\beta}_{11}$	0.95	0.94	0.96	0.94	0.94
				$\hat{\beta}_{12}$	0.94	0.93	0.93	0.94	0.94
		High (B)	IPW	$\hat{\beta}_{11}$	0.94	0.94	0.95	0.94	0.94
				$\hat{\beta}_{12}$	0.94	0.94	0.92	0.94	0.93
			GR	$\hat{\beta}_{11}$	0.95	0.95	0.95	0.93	0.94
				$\hat{\beta}_{12}$	0.94	0.93	0.92	0.94	0.94
	High	Low (C)	IPW	$\hat{\beta}_{11}$	0.94	0.95	0.94	0.94	0.94
				$\hat{\beta}_{12}$	0.94	0.94	0.93	0.94	0.94
			GR	$\hat{\beta}_{11}$	0.95	0.94	0.94	0.95	0.95
				$\hat{\beta}_{12}$	0.95	0.94	0.94	0.95	0.94
		High (D)	IPW	$\hat{\beta}_{11}$	0.96	0.94	0.94	0.94	0.95
				$\hat{\beta}_{12}$	0.95	0.93	0.93	0.94	0.94
			GR	$\hat{\beta}_{11}$	0.95	0.95	0.94	0.94	0.94
				$\hat{\beta}_{12}$	0.95	0.94	0.93	0.94	0.94

Supplemental Table S9: Coverage probabilities of estimated 95% confidence intervals for the simulation study for the two outcomes and two predictors (2O2P) scenario.

**Scen-ario**	**Corr level**	**Error level**	**Estim-ator**		**Strat 1: Case-control**	**Strat 2: Simult-aneous**	**Strat 3: Sequential**	**Strat 5: A-optimal**
2O2P	Moderate	Low (A)	IPW	$\hat{\beta}_{11}$	0.96	0.95	0.95	0.95
				$\hat{\beta}_{12}$	0.94	0.94	0.94	0.95
				$\hat{\beta}_{21}$	0.95	0.95	0.94	0.94
				$\hat{\beta}_{22}$	0.95	0.94	0.91	0.93
			GR	$\hat{\beta}_{11}$	0.95	0.95	0.95	0.94
				$\hat{\beta}_{12}$	0.95	0.95	0.94	0.94
				$\hat{\beta}_{21}$	0.95	0.95	0.95	0.94
				$\hat{\beta}_{22}$	0.95	0.93	0.93	0.94
		High (B)	IPW	$\hat{\beta}_{11}$	0.94	0.94	0.95	0.94
				$\hat{\beta}_{12}$	0.94	0.94	0.93	0.94
				$\hat{\beta}_{21}$	0.95	0.95	0.94	0.94
				$\hat{\beta}_{22}$	0.94	0.94	0.90	0.93
			GR	$\hat{\beta}_{11}$	0.95	0.95	0.95	0.95
				$\hat{\beta}_{12}$	0.95	0.93	0.94	0.94
				$\hat{\beta}_{21}$	0.95	0.95	0.94	0.95
				$\hat{\beta}_{22}$	0.95	0.94	0.91	0.93

Supplemental Table S10: Empirical variances ($\times 10^3$) for the A-optimal strategy (Strategy 5) under varying priority weights in the two outcomes of interest and one predictor scenario with low measurement error and low correlation (2O-A).

a_1	a_2	Var($\hat{\beta}_{11}$)	Var($\hat{\beta}_{12}$)
0.00	1.00	2.96	1.94
0.25	0.75	2.14	2.03
0.50	0.50	1.93	2.13
0.75	0.25	1.90	2.19
1.00	0.00	1.89	3.73

Supplemental Table S11: Empirical variance ($\times 10^3$) for each strategy under Scenario 2O: Two outcomes of interest in the supplemental scenario where the phase 2 size is $n = 500$.

**Corr level**	**Error level**	**Estim-ator**		**Strat 1: Case-control**	**Strat 2: Simult-aneous**	**Strat 3: β_{12} last**	**Strat 4: β_{11} last**	**Strat 5: A-optimal**
Low	Low (A)	IPW	$\mathrm{Var}(\hat\beta_{11})$	11.50	5.26	5.99	4.93	5.03
			$\mathrm{Var}(\hat\beta_{12})$	11.54	5.25	4.98	6.22	5.20
		GR	$\mathrm{Var}(\hat\beta_{11})$	3.94	3.39	3.76	3.00	3.35
			$\mathrm{Var}(\hat\beta_{12})$	5.01	4.23	3.72	4.39	3.83
	High (B)	IPW	$\mathrm{Var}(\hat\beta_{11})$	12.34	10.02	11.17	10.86	10.09
			$\mathrm{Var}(\hat\beta_{12})$	11.34	8.08	8.55	9.61	8.12
		GR	$\mathrm{Var}(\hat\beta_{11})$	9.16	9.12	11.09	9.28	9.13
			$\mathrm{Var}(\hat\beta_{12})$	8.18	7.98	7.68	8.65	7.89
High	Low (C)	IPW	$\mathrm{Var}(\hat\beta_{11})$	15.26	8.62	9.25	7.81	8.66
			$\mathrm{Var}(\hat\beta_{12})$	15.60	7.43	6.33	8.63	6.90
		GR	$\mathrm{Var}(\hat\beta_{11})$	6.16	5.48	6.22	5.27	5.41
			$\mathrm{Var}(\hat\beta_{12})$	6.76	5.22	4.75	5.85	5.18
	High (D)	IPW	$\mathrm{Var}(\hat\beta_{11})$	16.97	16.53	17.20	16.35	15.30
			$\mathrm{Var}(\hat\beta_{12})$	14.14	9.62	9.49	11.82	10.30
		GR	$\mathrm{Var}(\hat\beta_{11})$	13.44	15.03	15.93	15.41	14.84
			$\mathrm{Var}(\hat\beta_{12})$	10.90	9.68	9.28	10.93	10.15

Supplemental Table S12: Empirical variance ($\times 10^3$) for each strategy under Scenario 2P: Two predictors of interest in the supplemental scenario where the phase 2 size is $n = 500$.

**Corr level**	**Error level**	**Estim-ator**		**Strat 1: Case-control**	**Strat 2: Simult-aneous**	**Strat 3: β_{22} last**	**Strat 4: β_{12} last**	**Strat 5: A-optimal**
Low	Low (A)	IPW	$\mathrm{Var}(\hat\beta_{12})$	13.66	9.25	9.33	8.66	9.25
			$\mathrm{Var}(\hat\beta_{22})$	15.66	11.28	12.06	11.58	11.64
		GR	$\mathrm{Var}(\hat\beta_{12})$	7.81	6.57	6.59	6.83	6.55
			$\mathrm{Var}(\hat\beta_{22})$	11.02	10.09	10.53	10.43	10.19
	High (B)	IPW	$\mathrm{Var}(\hat\beta_{12})$	13.83	11.90	12.03	12.45	11.63
			$\mathrm{Var}(\hat\beta_{22})$	16.33	14.10	14.07	14.77	13.88
		GR	$\mathrm{Var}(\hat\beta_{12})$	10.46	10.84	10.16	10.70	10.04
			$\mathrm{Var}(\hat\beta_{22})$	13.27	13.11	13.24	13.53	12.51
High	Low (C)	IPW	$\mathrm{Var}(\hat\beta_{12})$	23.02	17.55	18.81	18.36	17.82
			$\mathrm{Var}(\hat\beta_{22})$	25.24	20.45	20.67	20.29	19.97
		GR	$\mathrm{Var}(\hat\beta_{12})$	15.04	14.00	13.93	14.12	13.43
			$\mathrm{Var}(\hat\beta_{22})$	18.77	17.75	18.10	17.95	17.18
	High (D)	IPW	$\mathrm{Var}(\hat\beta_{12})$	25.07	21.46	22.27	22.87	22.38
			$\mathrm{Var}(\hat\beta_{22})$	29.23	23.86	24.99	23.96	24.70
		GR	$\mathrm{Var}(\hat\beta_{12})$	20.41	20.29	19.97	20.34	19.62
			$\mathrm{Var}(\hat\beta_{22})$	24.89	22.39	23.31	23.26	21.87

Supplemental Table S13: Coverage probabilities of estimated 95% confidence intervals for the simulation study for the two outcomes (2O) and two predictors (2P) scenarios in the supplemental scenario where the phase 2 size is $n = 500$.

**Scen-ario**	**Corr level**	**Error level**	**Estim-ator**		**Strat 1: Case-control**	**Strat 2: Simult-aneous**	**Strat 3: Sequential order 1**	**Strat 4: Sequential order 2**	**Strat 5: A-optimal**
2O	Low	Low (A)	IPW	$\hat{\beta}_{11}$	0.94	0.94	0.94	0.94	0.95
				$\hat{\beta}_{12}$	0.95	0.94	0.93	0.94	0.94
			GR	$\hat{\beta}_{11}$	0.95	0.94	0.95	0.94	0.94
				$\hat{\beta}_{12}$	0.95	0.93	0.92	0.94	0.93
		High (B)	IPW	$\hat{\beta}_{11}$	0.95	0.94	0.94	0.90	0.93
				$\hat{\beta}_{12}$	0.95	0.93	0.92	0.94	0.93
			GR	$\hat{\beta}_{11}$	0.95	0.94	0.93	0.91	0.94
				$\hat{\beta}_{12}$	0.95	0.93	0.92	0.93	0.93
	High	Low (C)	IPW	$\hat{\beta}_{11}$	0.94	0.94	0.94	0.93	0.94
				$\hat{\beta}_{12}$	0.95	0.93	0.95	0.94	0.94
			GR	$\hat{\beta}_{11}$	0.94	0.94	0.94	0.92	0.94
				$\hat{\beta}_{12}$	0.95	0.93	0.93	0.93	0.93
		High (D)	IPW	$\hat{\beta}_{11}$	0.95	0.93	0.93	0.91	0.93
				$\hat{\beta}_{12}$	0.95	0.94	0.94	0.94	0.94
			GR	$\hat{\beta}_{11}$	0.95	0.93	0.94	0.91	0.92
				$\hat{\beta}_{12}$	0.95	0.93	0.92	0.92	0.91
2P	Low	Low (A)	IPW	$\hat{\beta}_{11}$	0.94	0.93	0.94	0.94	0.95
				$\hat{\beta}_{12}$	0.94	0.94	0.92	0.94	0.93
			GR	$\hat{\beta}_{11}$	0.95	0.93	0.94	0.94	0.94
				$\hat{\beta}_{12}$	0.94	0.94	0.92	0.94	0.93
		High (B)	IPW	$\hat{\beta}_{11}$	0.95	0.94	0.94	0.93	0.93
				$\hat{\beta}_{12}$	0.95	0.93	0.92	0.93	0.93
			GR	$\hat{\beta}_{11}$	0.95	0.94	0.94	0.93	0.94
				$\hat{\beta}_{12}$	0.95	0.93	0.92	0.94	0.93
	High	Low (C)	IPW	$\hat{\beta}_{11}$	0.95	0.94	0.94	0.93	0.94
				$\hat{\beta}_{12}$	0.95	0.93	0.93	0.95	0.94
			GR	$\hat{\beta}_{11}$	0.95	0.94	0.94	0.93	0.94
				$\hat{\beta}_{12}$	0.95	0.94	0.93	0.94	0.94
		High (D)	IPW	$\hat{\beta}_{11}$	0.95	0.93	0.93	0.93	0.93
				$\hat{\beta}_{12}$	0.94	0.93	0.92	0.94	0.92
			GR	$\hat{\beta}_{11}$	0.95	0.93	0.93	0.93	0.94
				$\hat{\beta}_{12}$	0.94	0.93	0.93	0.93	0.93

References

Amorim, G., R. Tao, S. Lotspeich, P. A. Shaw, T. Lumley, and B. E. Shepherd (2021). Two-phase sampling designs for data validation in settings with covariate measurement error and continuous outcome. *Journal of the Royal Statistical Society Series A: Statistics in Society 184*(4), 1368–1389.

Binder, D. A. (1983). On the variances of asymptotically normal estimators from complex surveys. *International Statistical Review 51*, 279–292.

Breslow, N. E., T. Lumley, C. M. Ballantyne, L. E. Chambless, and M. Kulich (2009). Improved Horvitz–Thompson estimation of model parameters from two-phase stratified samples: applications in epidemiology. *Statistics in Biosciences 1*, 32–49.

Han, K., P. A. Shaw, and T. Lumley (2021). Combining multiple imputation with raking of weights: An efficient and robust approach in the setting of nearly true models. *Statistics in Medicine 40*(30), 6777–6791.

Kluger, D. M. and S. Bates (2026). M-estimation under two-phase multiwave sampling with applications to prediction-powered inference. *arXiv preprint arXiv:2602.16933*.

Lumley, T. (2004). Analysis of complex survey samples. *Journal of statistical software 9*, 1–19.

Lumley, T. (2011). *Complex surveys: a guide to analysis using R.* John Wiley & Sons.

Oh, E. J., B. E. Shepherd, T. Lumley, and P. A. Shaw (2021). Raking and regression calibration: Methods to address bias from correlated covariate and time-to-event error. *Statistics in Medicine 40*(3), 631–649.

Särndal, C. E., B. Swensson, and J. Wretman (2003). *Model assisted survey sampling.* Springer Science & Business Media.

Shepherd, B., K. Han, T. Chen, A. Bian, S. Pugh, S. Duda, T. Lumley, W. Heerman, and P. Shaw (2023). Multiwave validation sampling for error-prone electronic health records. *Biometrics 79*(3), 2649–2663.

Yang, J., T. Lumley, B. Shepherd, and P. Shaw (2025). Optimum allocation for adaptive multi-wave sampling in R: The R package optimall. *Journal of Statistical Software 114*, 1–31.
